# Unveiling the Bioactive Compounds and Therapeutic Potential of *Russula*: A Comprehensive Review

**DOI:** 10.3390/jof11050341

**Published:** 2025-04-27

**Authors:** Jingya Yang, Samantha C. Karunarathna, Nimesha Patabendige, Entaj Tarafder, Dengji Lou, Yuanqing Zhou, Kalani Hapuarachchi

**Affiliations:** 1 College of Chemistry Biology and Environment, Yuxi Normal University, Yuxi 653100, China; yangjingya@yxnu.edu.cn (J.Y.); loudengji@yxnu.edu.cn (D.L.); yqzhou@yxnu.edu.cn (Y.Z.); 2 Center for Yunnan Plateau Biological Resources Protection and Utilization, College of Biology and Food Engineering, Qujing Normal University, Qujing 655011, China; samanthakarunarathna@gmail.com; 3 School of Medical, Molecular and Forensic Sciences, Murdoch University, Murdoch, WA 6150, Australia; nimesha.patabendige@gmail.com; 4 Department of Plant Pathology, College of Agriculture, Guizhou University, Guiyang 550025, China; entajmycology@gmail.com; 5 College of Biodiversity Conservation, Southwest Forestry University, Kunming 650224, China

**Keywords:** Basidiomycetes, medicinal properties, mushrooms, traditional medicine

## Abstract

*Russula*, a genus of Basidiomycetes with considerable taxonomic diversity, holds significant potential in both traditional and modern medicinal practices. This comprehensive review explores the bioactive compounds identified in various *Russula* species, detailing their characterization, structural elucidation, and classification. The medicinal properties of these fungi are examined, with a focus on their antioxidant, anti-inflammatory, and immunomodulatory effects, supported by both historical usage and contemporary preclinical pharmacological research. The review also highlights emerging biotechnological applications including environmental remediation, antimicrobial agents, and functional food development. Safety and toxicological considerations are evaluated to provide a balanced perspective on the medicinal use of *Russula*. The review concludes by summarizing the key findings and emphasizing the importance of *Russula* in both traditional medicine and future clinically validated innovations.

## 1. Introduction

The family Russulaceae [1] comprises a diverse group of fungi, including forms such as agaricoid, secotioid, pleurotoid, and gasteroid, which are well known for forming ectomycorrhizal relationships with a variety of host plants and have a worldwide distribution [2,3,4,5,6,7]. Molecular phylogenetic studies confirm that Russulaceae is a monophyletic group consisting of seven genera: *Russula*, *Lactarius*, *Lactifluus*, *Boidinia*, *Multifurca Gloeopeniophorella*, and *Pseudoxenasma* [8,9,10]. Among these, *Russula* Pers. is the most dominant genus [11,12]. There are 3218 published species names under *Russula* (www.indexfungorum.org, accessed on 8 April 2025), reflecting the vast diversity and ongoing discovery of new species within this genus. This genus is characterized by its fleshy fruiting bodies, colorful and fragile caps, amyloid warty basidiospores, abundant sphaerocysts in the trama, and lack of latex and clamp connections in the hyphae [3,13,14,15,16]. *Russula* species are commonly found in tropical and subtropical evergreen forests but have a broad distribution from Western Europe to North America, Africa, and various Asian countries, especially in China and India [15,17,18,19,20,21,22,23,24,25,26,27,28,29,30,31,32,33,34,35,36,37].

The genus *Russula* plays a significant ecological role as ectomycorrhizal fungi that form symbiotic relationships with trees and contribute to nutrient cycling in forest ecosystems [13,38,39,40,41,42,43]. Traditional taxonomic classification has been based on morphological characteristics such as cap color, gill attachment, and spore print color, as well as the chemical reactions of different parts of the fruiting body, which are important taxonomic characters in *Russula* [44,45,46,47]. However, these features exhibit high variability and overlap between species, creating challenges for accurate identification [48,49]. Recent progress in molecular phylogenetics has provided deeper insights into the evolutionary relationships within *Russula*, leading to species reclassifications and the discovery of previously cryptic species [9,50,51,52]. The incorporation of molecular data has greatly improved the understanding of genetic diversity, evolutionary history, and ecological functions in this genus [53,54,55,56,57,58].

**Figure 1:**
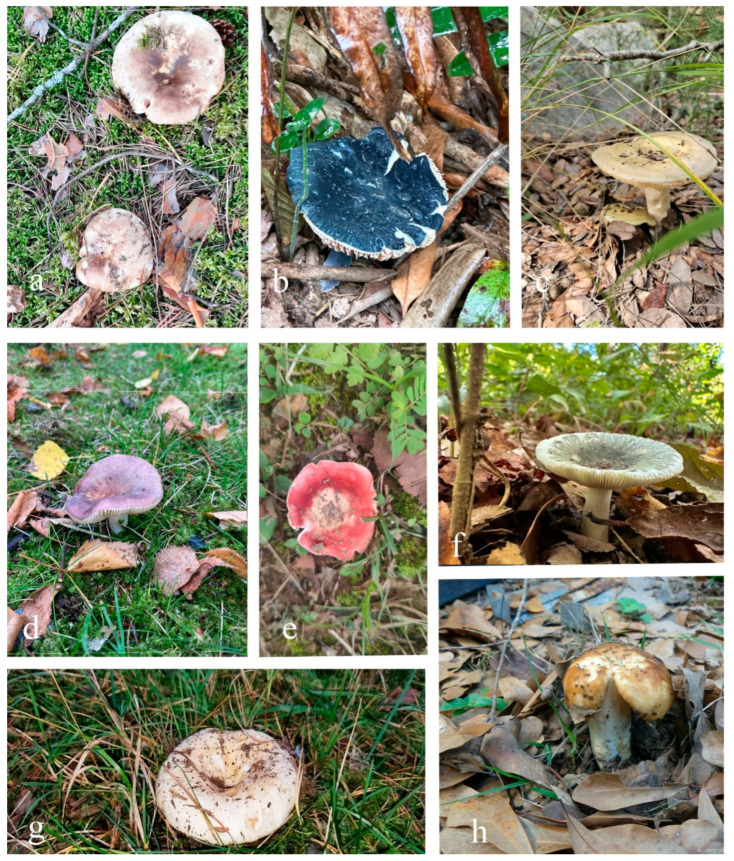
*Russula* species (**a**) *Russula adusta* (**b**) *R. albonigra* (**c**) *R. alutacea* (**d**) *R. cyanoxantha* (**e**) *R. griseocarnosa* (**f**) *R. virescens* (**g**) *R. delica* (**h**) *R. foetens* (https://www.inaturalist.org/; accessed on 10 March 2025, the images are used under the license Attribution Non-Commercial-No Derivs 4.0).

Many *Russula* species are highly valued for their edibility, with some, such as R. cyanoxantha and *R. virescens*, being globally renowned; in contrast, others, including *R. griseocarnosa* and *R. adusta*, hold regional commercial importance [59,60,61,62] (Figure 1, Table 1). *Russula* have long held historical and cultural significance, especially in Europe, Asia, and Eastern Europe, where they have been used in traditional cuisine and medicine for centuries [63,64,65,66,67]. China has a long history of using various *Russula* species as traditional foods and medicines [68,69,70,71,72]. Valued for their unique flavors, they are integral to regional dishes and are also employed in treating ailments such as indigestion and inflammation. In addition, they are nutritionally rich, providing carbohydrates, proteins, fiber, vitamins, and essential minerals, further contributing to their dietary and medicinal significance [73,74,75,76]. Extracts from these mushrooms have been shown to reduce inflammation and oxidative stress in various studies. These medicinal extracts are often utilized in various forms, including teas, tinctures, and capsules [77]. Beyond culinary and medicinal uses, these mushrooms have been symbolically significant in various cultures, representing fertility and prosperity, and have inspired artistic expressions and folklore due to their distinctive appearance [78]. *Russula* are processed into dietary supplements and nutraceuticals for their bioactive compounds, which offer immune-boosting and anti-aging benefits due to their rich content of antioxidants, vitamins, and minerals. In addition, *Russula* extracts are used in cosmetic products for their antioxidant properties, helping protect the skin from oxidative stress, improve hydration, reduce wrinkles, and enhance elasticity, making them valuable in anti-aging skincare [70,79].

The bioactive compounds in *Russula* demonstrate significant therapeutic potential due to their distinctive secondary metabolite profiles. These characteristic combinations of compounds—while sharing structural motifs with other fungi—exhibit marked antioxidant, anti-inflammatory, antimicrobial, and immunomodulatory activities, underscoring the pharmacological importance of this genus [86,141,142,143,144]. As research continues, these compounds are being explored for their potential in drug discovery and natural product development, with advancements in biotechnological techniques further enhancing the ability to harness these properties for various applications. While *Russula* mushrooms are generally safe for consumption, it is important to consider potential toxicity, as some species contain harmful compounds [145,146]. Toxicological studies are essential to establish guidelines for safe use, especially as these mushrooms are increasingly integrated into modern medicine and biotechnology. Ongoing research into their medicinal potential, combined with a focus on safety, ensures that *Russula* will continue to be valuable both in traditional practices and in innovative scientific applications.

This comprehensive understanding of *Russula*, encompassing their taxonomical classification, ecological significance, and diverse applications, underscores their multifaceted value. Their integration into traditional and modern practices highlights both their cultural importance and potential in scientific research. As exploration into their bioactive compounds and medicinal properties continues, *Russula* stands as a vital resource for future biotechnological advancements and therapeutic developments.

## 2. Bioactive Compounds and Beneficial Medicinal Properties of *Russula*

**Figure 2:**
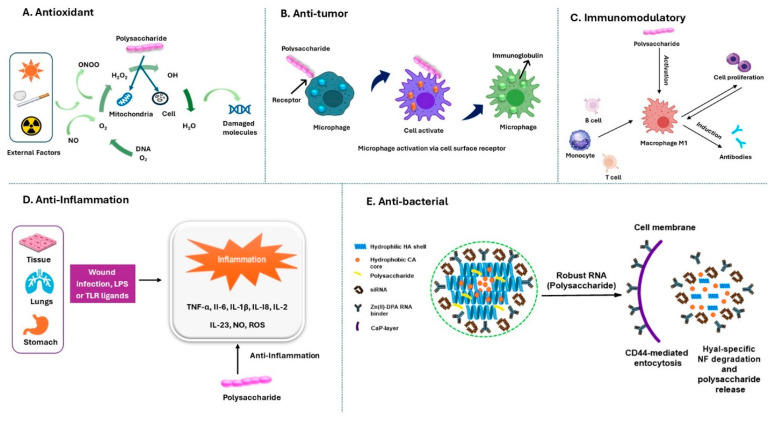
Bioactive properties and mechanism of polysaccharides from *Russula* [99]. (**A**) Antioxidant: polysaccharides scavenge ROS, protecting cells from oxidative damage. (**B**) Anti-tumor: they activate macrophages, enhancing the immune response. (**C**) Immunomodulatory: they stimulate immune cells, boosting antibody production and cell proliferation. (**D**) Anti-inflammatory: they reduce cytokines (TNF-α, IL-6, and IL-1β) and oxidative stress. (**E**) Anti-bacterial: they facilitate bacterial RNA targeting and membrane degradation via CD44-mediated endocytosis.

**Figure 3:**
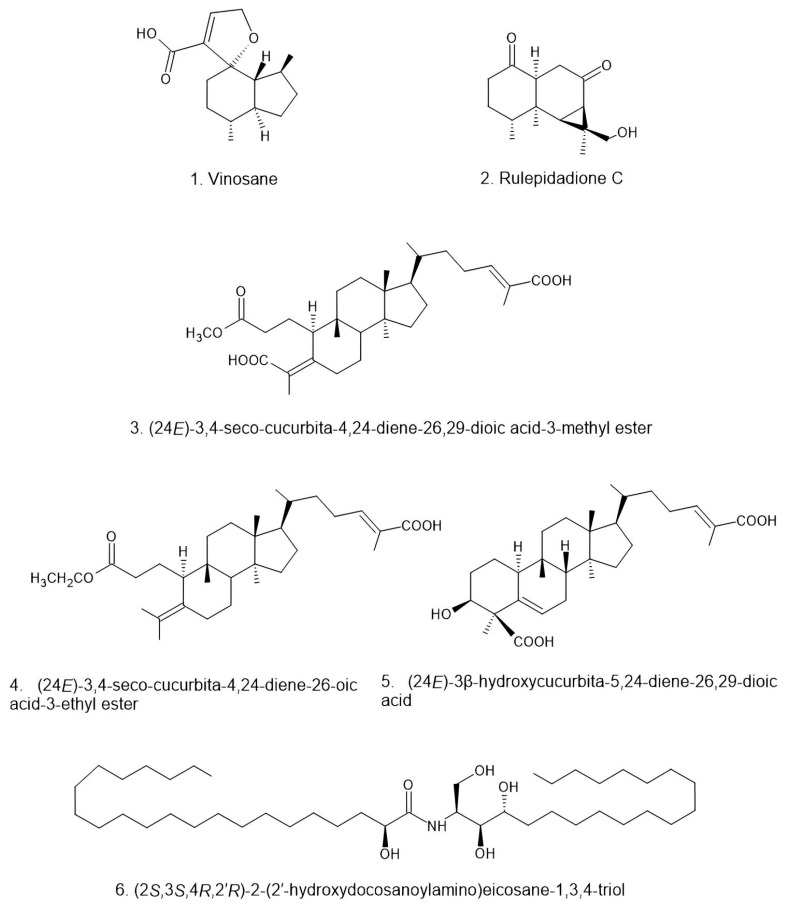

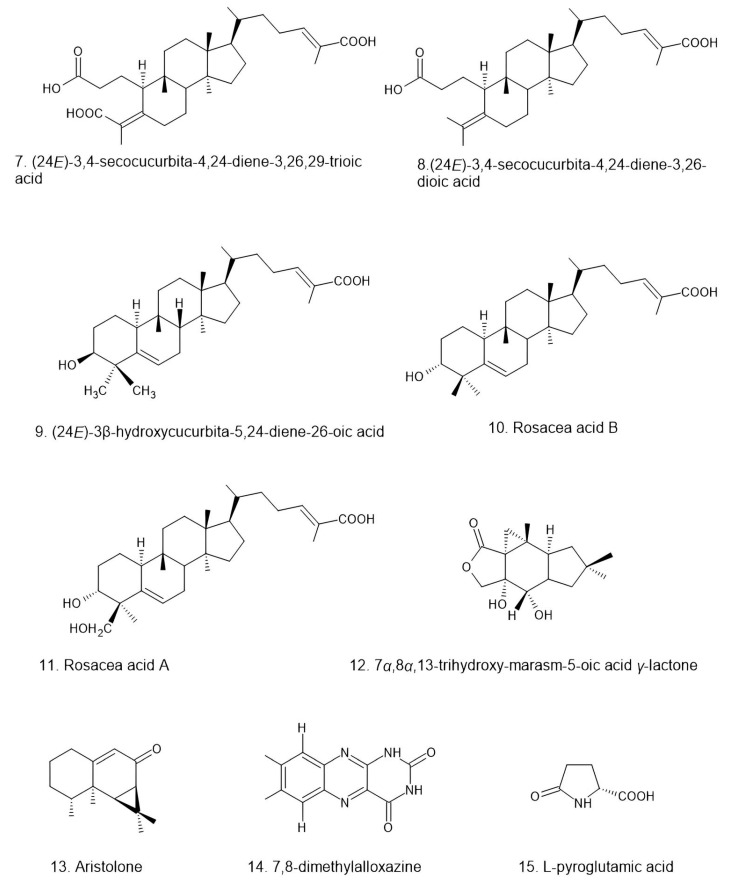
Structural diversity of triterpenoids isolated from *Russula*, including seco-cucurbitane and lanostane derivatives [148]. These triterpenoids exhibit structural variations in oxidation patterns, side-chain modifications, and ring configurations, which may contribute to their diverse bioactivities.

*Russula* is enriched with several species like *R. alboareolata*, *R. alutacea*, *R. helios*, *R. medullata*, *R. monspeliensis*, and *R. virescens*, all of which are widely consumed as foods [83,84,85]. In addition to their edibility, species of *Russula* are also used as traditional medicines for the treatment of various diseases like fever (*R. cyanoxantha* and *R. nobilis*), wound healing (*R. luteotacta*), treating gastritis and high blood pressure (e.g., *R. delica* and *R. parazurea*), and even in skin cancer (*R. acrifolia*) [99]. Moreover, some *Russula* species have also been traditionally used as tonics such as *R. acrifolia*, *R. cyanoxantha*, *R. delica*, *R. luteotacta*, *R. nobilis*, and *R. parazurea* [80]. In addition, *R. luteotacta* is also used as a sleep-promoting agent [80]. Apart from all this usefulness, there are reports about side effects and toxicities of some *Russula* species as well [127]. *Russula densifolia*, *R. fragtissima*, and *R. rosacea* can cause gastroenteritis, while *R. olivacea* can cause nausea, vomiting, and diarrhea [100]. *Russula subnigricans* can cause rhabdomyolysis, severe electrolyte disturbance (hypocalcemia), respiratory failure, acute renal failure, pulmonary edema, ventricular tachycardia, and circulatory shock [127]. Few species of *Russula* are poisonous, like *R. emetica* and *R. nigricans* [101]. Moreover, the consumption of *R. risigallina*, *R. olivacea*, and *R. velenovskyi* may poseharmful effects due to exceedingly elevated levels of Cr and Cd, as compared to reference safety limits [147]. In spite of the large number of taxa, the secondary metabolites of *Russula* have not been well researched. However, in the following part of this paper, we discuss various bioactive compounds (Figure 2 and Figure 3) produced by *Russula* species and their beneficial medicinal properties.

### 2.1. Bioactive Compounds

#### 2.1.1. Polysaccharides

##### *Russula* *alatoreticula*

A water-soluble, polysaccharide-rich extract (Rusalan) was isolated from the dried basidiocarps of *R. alatoreticula*, showing a triple helical conformation with glucose as the main monosaccharide. Rusalan exhibited strong antioxidant activity and effectively inhibited the growth of *Staphylococcus aureus* and *Bacillus subtilis*. In addition, it demonstrated immune-stimulatory effects in mouse macrophage cells [105]. The anti-oxidant activity of Rusalan has been demonstrated through its significant potential in scavenging hydroxyl, superoxide, and DPPH radicals, its metal ion chelating ability, and its capacity to donate electrons [105]. The extract also inhibits the growth of certain pathogenic bacteria [107]. Moreover, the crude polysaccharide showed high immune-modulatory activity without cytotoxicity [105]. Hence, Rusalan could be used as an ingredient for pharmaceutical use against free radicals, antibiotic-resistant pathogens, and hypo-immunity [105]. A β-glucan-enriched fraction, RualaCap, from the residue of *R. alatoreticula* after hot-water extraction, exhibited strong antioxidant and immune-enhancing properties. RualaCap demonstrated significant radical scavenging, chelating ability, and increased macrophage activity, along with the activation of key immune-related genes. These findings suggest its potential as a potent nutraceutical for immune stimulation [87]. *Russula alatoreticula* is rich in phenolic compounds and ascorbic acid, contributing to its strong antioxidant, antibacterial, and anticancer properties. The methanol extract demonstrated significant free radical quenching, Fe^2+^ ion chelation, and antibacterial activity, as well as apoptosis induction in Hep3B cells via the mitochondrial pathway. This suggests *R. alatoreticula* has substantial potential as a natural supplement for combating free radicals, pathogens, and hepatocellular carcinoma, with applications in food safety [149].

##### *Russula* *albonigra*

The water-soluble polysaccharide fraction of *R. albonigra* was found to inhibit the replication of intracellular amastigotes in macrophages dose-dependently and revealed its anti-proliferating effect [81]. A water-soluble β-glucan was isolated from the alkaline extract of the ectomycorrhizal edible mushroom *R. albonigra*. This compound showed in vitro macrophage activation via NO production and splenocyte and thymocyte proliferation. Furthermore, it has exhibited potent antioxidant activities [150]. Moreover, a water-soluble glucan [88] and a heteroglycan were isolated from the aqueous extract of *R. albonigra* [89]. These compounds showed in vitro macrophage activation via NO production, splenocytes, and thymocyte proliferation [89].

##### *Russula* *alutacea*

*Russula alutacea* polysaccharides are alkali-soluble but water-insoluble, limiting their use. Acetylation improved their solubility and enhanced antioxidant activity. Both acetylated and water-soluble polysaccharides, along with vitamin C, showed strong superoxide anion scavenging, with acetylated polysaccharides having the best DPPH radical-scavenging ability [151]. *Russula alutacea* polysaccharides were chemically modified to enhance water solubility and biological activity. The sulfated polysaccharides showed the highest scavenging activity for hydroxyl radicals, while vitamin C was more effective against superoxide anions and DPPH radicals [152]. A purified polysaccharide isolated from *R. alutacea* significantly reduced cell morphological changes and nitric oxide (NO) production in LPS-induced RAW 264.7 cells, both extracellularly and intracellularly. It down-regulated NF-κB, iNOS, and COX-2 expression, and alleviated oxidative stress and mitochondrial dysfunction through MAPK signaling pathways. Thus, *R. alutacea* is potentially a resource for protecting against inflammatory and oxidative damage [85].

##### *Russula* *griseocarnosa*

Polysaccharides from *R. griseocarnosa* demonstrate significant scavenging effects on superoxide anions and hydroxyl radicals, contributing to their strong antioxidative properties [150]. Alcohol extracts from *R. griseocarnosa* fruit bodies have been tested and shown to possess antibacterial properties against *Escherichia coli* and *S. aureus* [153]. RGP2, a polysaccharide from *R. griseocarnosa*, improved immune function in cyclophosphamide-induced immunosuppressed mice. With a molecular weight of 11.82 kDa, RGP2 enhanced spleen health, altered gut microbiota and serum metabolites, and boosted immune responses by affecting macrophages and T cells via the AKT/mTOR pathway [154]. Polysaccharides from *R. griseocarnosa* fruit bodies effectively neutralize hydroxyl and superoxide radicals. In vitro studies demonstrate that these polysaccharides significantly inhibit the proliferation of HeLa and SiHa cancer cells and enhance the phagocytic activity of peritoneal macrophages in mice, which, in turn, boosts the secretion of NO and cytokine IL-6, showcasing robust immunomodulatory properties [155]. Polysaccharide from *R. griseocarnosa* (PRG1-1) was found to enhance macrophage activation by increasing iNOS, COX-2, nitric oxide, and cytokine production. These effects are mediated through the NF-κB and MAPK signaling pathways, highlighting its potential as an immunomodulator [156].

RGP1, a polysaccharide from *R. griseocarnosa*, was shown to improve hematopoietic function in K562 cells. In mice with cyclophosphamide-induced hematopoietic dysfunction, RGP1 reduced bone-marrow damage, increased long-term hematopoietic stem cells, and regulated myeloid cells in the blood. It promoted CD4+ T-cell differentiation without affecting other immune cells. RGP1’s benefits were linked to CD4+ T-cell activation and the Janus kinase/STAT3 pathway, supporting its potential for treating hematopoietic dysfunction [157]. RGP2, a polysaccharide from *R. griseocarnosa*, improved immune function in cyclophosphamide-induced immunosuppressed mice. With a molecular weight of 11.82 kDa, RGP2 enhanced spleen health, altered gut microbiota and serum metabolites, and boosted immune responses by affecting macrophages and T-cells via the AKT/mTOR pathway [158]. RGP1, a galactan from *R. griseocarnosa*, alleviated CTX-induced hematopoietic dysfunction by reducing bone marrow damage, increasing stem cell numbers, and promoting CD4+ T-cell differentiation via the JAK/STAT3 pathway. Its structure features a 1,6-α-D-Galp backbone with O-3 methylation and α-L-Fucp branching [159]. Polysaccharides from wild *R. griseocarnosa* (PRG) exhibited antioxidant activities evidenced by reducing power to scavenge the DPPH, ABTS, hydroxyl radical, and superoxide radical [160,161]. PRG showed the activity of anti-cervical carcinoma cells Hela and Siha. Hence, PRG has good antioxidant and inhibitory activities against cervical carcinoma cells, and PRG could be developed as a novel natural functional food [162]. A novel polysaccharide, PRG1-1, was obtained from *R. griseocarnosa* sporocarp and the cytotoxicity effects of PRG1-1 on human cervical carcinoma are associated with the apoptotic pathway. Hence, *R. griseocarnosa* showed a promising potential of bioactive PRG1-1 as a natural agent to inhibit tumor cell proliferation in the treatment of cervical carcinoma [163]. PRG1-1 also has the ability to activate macrophages through the NF-κB and MAPK pathways, demonstrating significant immunomodulatory potential [156].

##### *Russula* *pseudocyanoxantha*

*Russula pseudocyanoxantha*, identified for its bioactive polysaccharides, was further explored by utilizing the solid remnants of conventional extraction processes, which contain therapeutic biopolymers. These were treated with cold alkali to yield a high-yield fraction (RP-CAP), characterized as having a β-glucan-rich carbohydrate backbone with a molecular weight of ~129.28 kDa. RP-CAP exhibited strong antioxidant and immune-boosting activities, promoting macrophage proliferation and inflammatory mediator synthesis through the TLR/NF-κB pathway. These findings suggest RP-CAP’s potential in functional foods and pharmaceuticals [120]. *Russula pseudocyanoxantha* has yielded a bioactive-rich fraction (RP-HAP) through extraction with a hot alkali solution. This β-glucan-enriched extract, with a molecular weight of approximately 111.25 kDa, demonstrated significant antioxidant activity and enhanced immune responses in RAW264.7 macrophage cells, indicating its potential for developing health-promoting pharmaceuticals [121].

##### *Russula* *virescens*

Water-insoluble (1→3)-β-D-glucan was firstly isolated from the fresh fruiting bodies of *Russula virescens* and then sulfated using sulfur trioxide–pyridine complex. The native (1→3)-β-D-glucan did not show anti-tumor activity, while the sulfated derivatives exhibited enhanced anti-tumor activities against sarcoma 180 tumor cells [92]. *Russula virescens* was studied for its water-soluble polysaccharides, RVP-1 and RVP-2. These non-triple-helix hetero-polysaccharides, composed of galactose, glucose, mannose, and fructose, exhibited promising antidiabetic, anticancer, and immunological activities. RVP-1 and RVP-2 were found to inhibit α-glucosidase and α-amylase activities, suppress cancer cell proliferation, and activate immune responses, providing a scientific basis for their potential therapeutic use [71]. Sulfonic acid groups (–SO3H) can be incorporated into polysaccharide molecules by replacing hydroxyl groups (–OH) through a process called sulfonation. This modification improves the interaction between sulfated polysaccharides from *R. virescens* and bacterial receptors, enhancing recognition and binding by altering the polysaccharide’s spatial structure and conformation [162]. Sulfated derivatives of the water-soluble polysaccharide from *R. virescens* (RVP) were prepared with varying degrees of substitution, resulting in compounds with altered molecular weights and conformations. These sulfated polysaccharides showed enhanced antioxidant, anticoagulant, antibacterial, and anti-tumor activities compared to the non-sulfated RVP, with SRVP1–25 exhibiting the strongest scavenging and anticoagulant effects, and SRVP1–20 demonstrating the best antibacterial and anti-tumor properties [164]. RVP, a water-soluble galactoglucomannan from *R. virescens*, was extracted using an alkali method and adopts a semi-rigid triple-helix structure. It has a low protein content (0.95%) and a molecular weight of 8.91 × 10^5^. RVP showed strong antioxidant activity by increasing cell viability, reducing malondialdehyde (MDA) levels, and enhancing antioxidant enzyme activity (SOD, CAT, and GSH-Px) in H_2_O_2_-induced oxidative stress models. These properties highlight its potential for health and wellness applications [165]. Furthermore, water-soluble polysaccharides from fruiting bodies of *R. virescens* could be developed as potential anti-oxidant, anti-coagulant, anti-bacterial, and anti-tumor agents for industrial and biomedical use [95].

##### *Russula* *vinosa*

Polysaccharides derived from *R. vinosa* have been demonstrated to boost lymphocyte activity and suppress the growth of SiHa cancer cells [131]. *Russula vinosa* exhibited higher β-glucan levels when assessed using the Congo red method compared to many other wild and commercial mushrooms, highlighting its potential as a valuable source of β-glucans for use in the food industry and medicinal purposes [132]. The water-soluble and alkali-soluble polysaccharides from *R. vinosa* showed antioxidant and hepatoprotective effects in vitro [133]. *Russula vinosa* has been consumed as a food in South China for a long time. Furthermore, plant growth regulator compounds were also isolated from the fruiting bodies of *R. vinosa* [134]. This mushroom contains β-glucans, which show immunomodulatory, anticancer, and antioxidant activities [132]. The acid extracts of *R. vinosa* demonstrated the highest ABTS (+) scavenging activity [166]. Furthermore, *R. vinosa* extracts inhibited the proliferation of HeLa and HepG2 cells in a dose-dependent manner. These results indicate that *R. vinosa* polysaccharides have potential antioxidant activity [166]. *Russula vinosa* was investigated for its anti-inflammatory effects using two polysaccharides (CA-S and CA-L) extracted with citric acid. Both showed similar structures but differed in substitution. CA-S and CA-L reduced disease activity index (DAI) values in ulcerative colitis mice by 36.84% and 31.58%, respectively. CA-S, with a higher molecular weight and more hydroxyl groups, was more effective in reducing inflammation through reactive oxygen species scavenging and the modulation of the Nrf2 and NFκB pathways [135]. *Russula vinosa* Lindblad is a carbohydrate-rich edible fungus. Two polysaccharides, RP-1 and RP-5, were extracted using KOH-graded extraction, with RP-5 showing a stronger immunomodulatory effect. A structural analysis revealed β-d-glucopyranose linkages, with RP-5 containing an additional mannopyranose residue. RP-5 enhanced macrophage phagocytosis by 121.04%, compared to 42.15% for RP-1, and both activated the NF-κB pathway, highlighting their potential as bioactive compounds [136].

##### *Russula* *adusta*, *R.* *aurea*, *R.* *delica*, *R.* *emetica*, and *R.* *senecis*

The polysaccharide named RAP was purified and characterized from *R. adusta*, showing a molecular weight of 5763 Da, with 80.03% total sugar, 0.17% protein, and 13.20% uronic acid. RAP primarily contained rhamnose, fucose, mannose, glucose, and galactose. RAP exhibited antioxidant activity, scavenging ·OH more effectively than O^2−^·, with activity increasing with concentration (0.25–8 mg/mL) [167]. Polysaccharides extracted from the mycelial culture of *R. aurea* inhibited the growth of sarcoma 180 and Ehrlich solid cancers by 70% and 60% in white mice, respectively [94]. Other research indicated that the water-insoluble (1–3)-β-D-glucan, isolated from the fresh fruiting bodies, did not show anti-tumor activity, whilst the sulfated derivatives exhibited enhanced anti-tumor properties [92]. The water-soluble polysaccharides also have antioxidant properties [93]. However, a study included *R. aurea* and *R. sanguinea*, but neither exhibited significant antioxidant, enzyme inhibitory, or antimutagenic activities [168].

*Leishmania donovani* is the causative agent of visceral leishmaniasis or kala azar in the Indian subcontinent [169]. The water-soluble polysaccharide fraction of *R. delica* was found to inhibit the replication of intracellular amastigotes in macrophages dose-dependently, and it showed its anti-proliferating effect [81]. Hence, this finding could be used in further phytochemical and pharmacological investigations in search of novel anti-leishmanial leads [81]. *Russula emetica* polysaccharides extracted via the ultrasound-assisted extraction method showed high anti-diabetic and antihypertensive activities [170]. Bioactive polysaccharides from *Rugibolutus extremiorientalis*, *R. emetica*, and *Phleobopus portentosus* were extracted using refluxing and ultrasound-assisted methods. *Russula extremiorientalis* polysaccharides had the highest antioxidant activity, while *R. emetica* showed the strongest antidiabetic and antihypertensive effects. The polysaccharides mainly contained carbohydrates, proteins, and glucose, with *R. extremiorientalis* and *R. emetica* having β-glycosidic linkages and *P. portentosus* having both α- and β-glycosidic linkages [170]. The crude polysaccharide Rusenan was extracted from *R. senecis*, and it can be used as a potent free-radical scavenger and murine macrophage stimulator [124]. Moreover, as for antioxidant activity, the crude polysaccharide exhibited strong potential in scavenging superoxide radicals, inhibiting OH generation, stabilizing DPPH, quenching ABTS radicals, inhibiting β-carotene bleaching, and demonstrating reducing power and Fe^2+^chelating ability [124]. Rusenan also exhibited strong immune-stimulation activities at low concentrations and initiated innate immunity by promoting macrophage proliferation, phagocytosis, morphological changes, NO release, ROS production, and the transcription of TLR-4, TLR-2, NF-κB, COX-2, iNOS. Its effects were comparable to those of standard antioxidant drugs [124]. The administration of *Russula* powder and polysaccharides has been found to significantly lower blood glucose, total cholesterol, triglycerides, and low-density lipoprotein levels in hyperglycemic and hyperlipidemic mice, demonstrating a dose-dependent effect and highlighting *Russula*’s potent hypoglycemic and lipid-lowering properties. Similarly, polysaccharide injections from *Russula* in hyperlipidemic rats led to a 45.2% reduction in total cholesterol compared to the control group [124]. The phytochemical analysis of *Cheimonophyllum candidissimus*, *Pleurotus* sp., *Russula* sp., and *Auricularia* sp. revealed bioactive compounds such as alkaloids, tannins, phenols, saponins, and flavonoids. These mushrooms also contained significant levels of protein, fats, fiber, carbohydrates, and essential minerals. However, high concentrations of heavy metals like cadmium, zinc, lead, and copper were found, which could be harmful if consumed in large amounts [171]. Table 2 lists polysaccharides from various *Russula* species and their associated biological activities.

#### 2.1.2. Terpenes

##### *Russula amarissima, R. brevipes,* and *R. cyanoxantha*

Four aristolane sesquiterpenes were isolated from the fruiting bodies of *R. amarissima* and *R. lepida* [91]. Also, a seco-cucurbitane triterpene, 3,4-secocucurbita-4, 24E-diene-3-hydroxy-26-carboxylic acid, was isolated from both species [91]. *Russula brevipes* produces triterpenoid compounds such as Lactarorufin A and Russulactarorufin [102]. The sphingolipid components of several higher fungi were investigated, with three new phytosphingosine-derived ceramides identified, including russulamide from *R. cyanoxantha* [172].

##### *R. delica, R. foetens,* and *R. japonica*

Protoilludane-type sesquiterpenoids have been isolated from *R. delica* [173]. Diethyl ether extract of the fruiting bodies of *R. delica* resulted in the isolation of a new norsesquiterpenoid, russulanorol, and eight known sesquiterpenoids, lactarorufin A, blennin C, furandiol, lactarorufin B, lactarolide A, 14-hydroxylactarolide A, 3-O-methyllactarolide B, and isolactarorufin [174,175]. *Russula foetens* is a poisonous mushroom that contains gastrointestinal irritants and several marasmane sesquiterpenes [176]. Methanol extract resulted in the isolation of a new marasmane sesquiterpene lactone named russulfoen, together with two known marasmane sesquiterpene lactones, 7α,8α,13-trihydroxy-marasm-5-oic acid γ-lactone [177] and 8α,13-dihydroxy-marasm-5-oic acid γ-lactone [176], one known ergosterol, (22E,24R)-5α,8α-epidioxyergosta-6,22-dien-3β-ol [178], as well as (1R,2R)-1-phenylpropane-1,2-diol [179]. *Russula foetens* was shown to produce the marasmane sesquiterpenes Lactapiperanol A and Lactapiperanol E [180]. A cytotoxic marasmane sesquiterpene, Russulfoen, was produced by *R. foetens* [179]. Illudoid sesquiterpenes were obtained from the fruiting body of *R. japonica* with neurite outgrowth-promoting activity [107]. Russujaponols A–F, illudoid sesquiterpenes isolated from the fruiting body of *Russula japonica*, exhibit notable neurite outgrowth-promoting activity and possess potential anticancer properties [106].

##### *Russula lepida, R. nobilis,* and *R. queletii*

Four aristolane sesquiterpenes were isolated from the fruiting bodies of *R. lepida* (=*Russula rosea*, www.indexfungorum.org, accessed on 26 March 2025) [91]. Three new triterpenoids and two new aristolane sesquiterpenoids were isolated from *R. lepida*. Notably, two of the compounds are the first naturally occurring seco-ring-A cucurbitane triterpenoids, while two others represent a rare type of aristolane sesquiterpenoids found among fungi [110,181]. From the extract of *R. lepida* fruiting bodies, which exhibit anti-tumor activity, four new cucurbitane-type triterpenoids were isolated: (24E)-3β-hydroxycucurbita-5,24-dien-26-oic acid, (24E)-3,4-seco-cucurbita-4,24-diene-3,26-dioic acid, (24E)-3,4-seco-cucurbita-4,24-diene-3,26,29-trioic acid, and lepidolide [181,182]. Fatty acid esters of velutinal, three new sesquiterpenoids, and Russulanobilines A–C, along with eight known compounds, were isolated from extracts of *R. nobilis* fruiting bodies [183]. These sesquiterpenes have unique structures for chemical defense machinery, which protects mushrooms against predators, parasites, and microorganisms [183]. Piperalol and piperdial, bioactive compounds isolated from *R. queletii* have shown potential in various biological activities, including antimicrobial and anticancer properties [122].

##### *Russula rosacea, R. sanguinaria, R. virescens,* and *R. vinosa*

Two new triterpenes, identified as rosacea acids A and B, with a similar structure, were extracted from the fruiting bodies of *R. rosacea* (Bull) Gray em. Fr. (*Russulaceae*) [184]. From *R. sanguinaria*, several compounds, including 15-hydroxyblennin A, blennin A, C, and D, lactarorufin A, piperalol, and vellerolactone, were identified [185]. Three new lactarane-type sesquiterpenoids, sangusulactones A-C, and two known ones, blennin A and 15-hydroxyblennin A, were isolated from the methanol extract of the fruiting bodies of the inedible mushroom *R. sanguinea* [123]. The phytochemical and bioactive profile of *R. virescens*, identifying 633 phytochemicals, including fatty acids, amino acids, polyphenols, and terpenoids, highlights its nutritional and medicinal potential. *Russula virescens* phytochemicals were also linked to cancer treatment pathways, revealing potential targets like HSP90AA1 and AKT3. While the work demonstrates the efficacy of multiomics techniques for exploring mushroom bioactivity, limitations include the lack of quantitative analysis and reliance on reversed-phase chromatography [143]. Five compounds, including triterpenoids, alcohols, and phenol (1R,2S)-1-phenylpropane-1,2-diol, isolactarorufin, lactarorufin A, 8α,13-dihydroxy-marasm-5-oic acid γ-lactone, and 7α,8α,13-trihydroxy-marasm-5-oic acid γ-lactone, were extracted from the fruiting bodies of *R. vinosa*. In a bioassay that tested plant growth regulatory activity on lettuce, all of these compounds demonstrated growth-regulating effects [186]. Fifteen compounds, including six new ones, were isolated and purified from *R. vinosa*; triterpenoids and sesquiterpenoids were its main chemical constituents. They showed potential anti-inflammatory effects [148]. Table 3 summarizes the triterpenoids isolated from various *Russula* species and their bioactivities

### 2.2. Other Bioactive Compounds and Beneficial Medicinal Properties of Russula

#### 2.2.1. *Russula aeruginea*, *R. albonigra*, *R. alnetorum*, *R. brevipes*, *R. fragrantissima*, *R. nobilis*, and *R. ochroleuca*

A nutrient analysis of the above species showed that the protein content varied between 28.12% and 42.86%, while the carbohydrate content ranged from 49.33% to 55% [90]. *Russula aeruginea* and *R. brevipes* exhibit significant antimicrobial and antioxidant activities, with *R. brevipes* showing strong antibacterial effects against *B. subtilis* and *R. aeruginea* demonstrating potent antifungal activity against *Fusarium equiseti*. The antioxidant potential of both species was confirmed through various assays, suggesting their potential as sources for developing new antimicrobial and antioxidant drugs [87]. An alcohol extract of *R. albonigra* showed protective effects against CCl_4_-induced liver damage in mice, normalizing liver enzymes and restoring antioxidant levels comparably to silymarin. These findings suggest its potential as a natural liver protectant [103].

#### 2.2.2. *Russula alboareolata*

The apoptotic activity of *R. alboareolata* ethanolic extract was tested on L929, HepG2, and HeLa cells. The extract induced significant apoptosis: 77.20% in L929 cells at 600 µg/mL, 73.69% in HeLa cells at 500 µg/mL, and 30.00% in HepG2 cells at 1000 µg/mL. Valinomycin was used as a positive control. The results suggest that the extract has potential apoptotic effects on both normal and cancer cells, indicating its possible application in dietary supplements or chemoprevention [188]. The ethanolic extract of *R. alboareolata* may be considered a natural supplement useful in the treatment of bacterial infections [189]. The anti-inflammatory effects of extracts from *Russula* species, including *R. alboareolata*, *R. medullata*, *R. virescens*, and *R. helios*, were studied. *Russula alboareolata* showed the strongest anti-inflammatory activity, inhibiting nitric oxide, prostaglandin E2, and COX-2, with minimal cytotoxicity, suggesting its potential as an effective anti-inflammatory agent [190].

#### 2.2.3. *Russula alatoreticula*

*Russula alatoreticula* methanol extract contains phenols, flavonoids, ascorbic acid, β-carotene, and lycopene [191]. Hence, it exhibited strong antioxidant activity through its ability to quench free radicals, chelate Fe^2+^ ions, and reduce components [192]. Furthermore, methanol extract showed effective antibacterial potential against six investigated microbes; *B. subtilis*, *E. coli*, *Klebsiella pneumoniae*, *Listeria monocytogenes*, *S. aureus*, and *S. typhimurium* [191]. The extract revealed promising anti-cancer properties as well [192]). Thus, *R. alatoreticula* can be utilized as a good source of natural supplement against free radicals, pathogenic bacteria, and hepatocellular carcinoma and, further, in the food safety industry [192]. *Russula alatoreticula* ethanolic extract, enriched with phenolics like pyrogallol and cinnamic acid, demonstrated strong antioxidant and antibacterial activities, particularly against Gram-positive bacteria. In addition, the extract showed potential in inhibiting Hep3B cell proliferation by inducing apoptosis through the intrinsic mitochondrial pathway [86].

#### 2.2.4. *Russula alveolata*, *Russula aruea*, *Russula aurora, Russula alveolata*, *Russula* cf. *Compressa*, *Russula flavobrunnea* var. *aurantioflava*, and *Russula ochrocephala*

The proximate compositions, minerals, and amino acid contents of *R. alveolata*, *Russula* cf. *compressa*, *R. flavobrunnea* var. *aurantioflava*, and *R. ochrocephala* from Burkina Faso were investigated. The analysis revealed the presence of various bioactive compounds, including volatile oil, sterols, triterpenes, carotenoids, and saponosides, along with essential amino acids such as phenylalanine, valine, threonine, isoleucine, methionine, leucine, and lysine [102]. A phytochemical analysis of the ethyl acetate fraction from *R. aruea* Pers fruiting bodies led to the identification of a new isolactarane, sesquiterpene, and 11 known compounds, including four sesquiterpenes, four sterols, one allitol, and two fatty acids. The sesquiterpenes in *R. aruea* may serve as chemotaxonomic markers [150]. In a study of phenolic acids in 26 mushroom species using HPLC–DAD, *R. aurora* was noted for its major phenolic compound, gallic acid. The method used also identified fumaric acid as the most abundant compound in many mushrooms and catechin hydrate in others. The study provided a standardized approach for phenolic acid profiling in mushrooms [96].

#### 2.2.5. *Russula brevipes*

The methanol extract of *R. brevipes* fruiting bodies and mycelia exhibited antioxidant activity with EC50 values of 0.89 mg/mL and 7.08 mg/mL, respectively, in the 2,2-diphenyl-1-picrylhydrazyl (DPPH) assay [193]. In a separate study, the methanol and water extracts of *R. brevipes* exhibited reducing power with 3.60 mg and 0.95 mg of GAE/g, respectively. The Ferredoxin-reducing substance (FRS) assay recorded IC50 values of 1.60 mg/mL and 1.80 mg/mL, and the extracts achieved 271% and 705% inhibition of lipid peroxidation, respectively [194]. *Russula brevipes* was studied for its bioactive compounds, revealing that decoction and infusion methods, due to higher phenolic content, demonstrated superior radical scavenging and metal ion chelating activities. This highlights *R. brevipes* as a promising natural source for reducing oxidative stress [195].

The ^1^H-NMR metabolomics profiling of six edible fungi, including *R. delica* and *R. brevipes*, confirmed the presence of essential amino acids, organic acids, nucleosides, and valuable nutraceuticals like betaine and carnitine. The analysis demonstrated how these *Russula* species, both phylogenetically related, could be grouped based on their chemical profiles, highlighting the nutritional and nutraceutical potential of these local foods [196].

#### 2.2.6. *Russula chloroides, Russula* cf. *foetentoides*, and *Russula foetens*

In *R. foetens* and *R. a* cf. *foetentoides* extracts, iron was the most abundant mineral in *R*. cf. *foetentoides*. Active secondary metabolites were identified, with gallic acid being the most concentrated phenolic compound. *Russula foetens* demonstrated the highest antibacterial and antifungal activities, particularly against *S. aureus* and *F. equiseti*. Both species exhibited significant antioxidant potential, with *R. foetens* showing the highest DPPH inhibition and reducing power, while *R*. cf. *foetentoides* had the highest ABTS inhibition, flavonoid, and total phenolic content [103]. Phenolic acids and flavonoids, like ferulic acid, gallic acid, and myricetin, found in *R. chloroides*, have been shown to enhance GST (glutathione S-transferase) activity [98].

#### 2.2.7. *Russula cutefracta* (=*R. cyanoxantha*) and *Russula cyanoxantha*

*Russula cutefracta* (=*R. cyanoxantha*, www.indexfungorum.org, accessed on 26 March 2025) inhibits degranulation in mast cells [197]. *Russula cyanoxantha* showed antifungal activity against *Microsporum canis* and antibacterial activity against *Pseudomonas putida* [198]. Ergosta-4, 6, 8(14), 22-tetraen-3-one (ergone), a bioactive steroid from *R. cyanoxantha*, has been demonstrated to possess cytotoxic and anti-proliferative activity towards HepG2 cells [199]. Lectins of this mushroom have significantly higher agglutination activity at 4 °C, as compared to room temperature [200]. A new phytosphingosine-type ceramide was isolated along with nine other compounds from extracts of the fruiting bodies of *R. cyanoxanotha* [172].

#### 2.2.8. *Russula delica*

The ethanolic extract of *R. delica* demonstrated antioxidant and antimicrobial activities, with inhibition values in the linoleic acid system increasing as the concentration increased. The extract contained 8.71 ± 0.56 μg mg^−1^ of total flavonoids and 47.01 ± 0.29 μg mg^−1^ of phenolic compounds, showing antibacterial but not anticandidal activity. These findings suggest that *R. delica* extracts could be valuable as antimicrobial and antioxidative agents in the food industry [201]. The methanolic extract of *R. delica* exhibited strong antioxidant activity, including reducing power and radical scavenging, surpassing some standard antioxidants like BHA, BHT, and α-tocopherol. The study also determined the total phenolic compounds, α-tocopherol, and β-carotene content in the extract, contributing to its potent antioxidant properties [202]. The ethanolic extract of *R. delica* shows antimicrobial activity against foodborne bacteria. The extract’s major phenolic component, catechin, was identified at 5.33 mg/L, and it demonstrated antioxidant properties, including 26% DPPH radical scavenging and 58% ferrous ion chelation. The extract also contained significant levels of total phenols, ascorbic acid, β-carotene, and lycopene [203]. *Russula delica* and *R. vesca* were among the 18 Portuguese wild mushrooms evaluated for their antioxidant properties. The study measured their radical-scavenging capacity, reducing power, and inhibition of lipid peroxidation, revealing that these mushrooms contain significant levels of antioxidants, including phenols and tocopherols. The research highlighted their potential for nutraceutical applications and emphasized the importance of managing and conserving these mushroom species [128].

*Russula delica* demonstrated the highest antioxidant activity among the mushrooms studied. This species also showed significant potential for use in developing safe antioxidants [204]. *Russula delica*, *R. lepida*, and *R. mustelina* mushrooms exhibit high levels of protein (38.08–38.52%), crude fiber (9.59–19.78%), carbohydrates (39.29–41.64%), ash (12.7–13.80%), and fat (4.06–5.70%). They are rich in potassium, phosphorus, calcium, and magnesium, with *R. delica* having the highest calcium and phosphorus content. Containing 18 amino acids, with glutamic acid and valine as predominant, their essential amino acid to total amino acid ratios range from 0.40 to 0.45, highlighting their high biological protein value [113].

The extract of *R. delica* was found to contain glycosaminoglycans, as identified using the dimethylmethylene blue (DMMB) dye-binding assay and UV-Vis spectrophotometry [205]. Fatty acids in *Pleurotus ostreatus* and *R. delica* were studied in total lipid, triacylglycerol, and phospholipid fractions, with palmitic, oleic, and linoleic acids as major components. Polyunsaturated fatty acids (PUFAs) were higher than monounsaturated (MUFAs) and saturated fatty acids (SFAs). Ethyl acetate extracts showed significant cytotoxicity against prostate carcinoma (PC-3) cells, with inhibition rates of 99.45–92.82% at 520–530 μg/mL and IC50 values of 274.53–297.77 μg/mL [206]. The ^1^H-NMR metabolomics profiling of six edible fungi, including *R. delica* and *R. brevipes*, confirmed the presence of essential amino acids, organic acids, nucleosides, and valuable nutraceuticals like betaine and carnitine. The analysis demonstrated how these *Russula* species, both phylogenetically related, could be grouped based on their chemical profiles, highlighting the nutritional and nutraceutical potential of these local foods [196].

#### 2.2.9. *Russula densifolia*, *Russula emetica* (M12), and *Russula fellea*

Extracts from *R. densifolia*, *R. violeipes*, and *R. cyanoxantha* showed strong antioxidant activities, including ABTS and DPPH radical scavenging, along with α-glucosidase and α-amylase inhibition. They also exhibited anti-inflammatory effects by inhibiting albumin denaturation and moderate antimicrobial activity. The ethanol extract of *R. violeipes* had notable cytotoxicity with an IC50 of 56.66 mg/mL, and the extracts contained significant levels of phenols and flavonoids [137]. *Russula emetica* (M12) showed multidrug resistance (MDR) reversal activity in paclitaxel-resistant P-glycoprotein (Pgp)-positive cancer cells, enhancing doxorubicin’s cytotoxicity in these cells. This suggests that compounds in *R. emetica* may be effective in reversing Pgp-associated drug resistance [207]. The anti-inflammatory and antimicrobial activities of 44 wild mushrooms from Rakuno Gakuen University in Japan were screened. Ten samples from five species, including *Naematoloma fasciculare*, *Cortinarius balteatocumatilis*, and *R. rosacea*, significantly reduced nitric oxide (NO) production, indicating strong anti-inflammatory effects [208].

#### 2.2.10. *Russula fragilis*, *R. fragrantissima,* and *R. gnathangensis*

The antimicrobial effects of protein extracts from rare mushrooms, including *R. fragilis*, *Ganoderma resinaceum*, and *Inocybe grammata*, were evaluated for their potential against common hospital pathogens. *Mycena pura* exhibited strong antagonism against *E. coli*. Unique protein patterns in exotic fungi further demonstrated significant inhibition of pathogens such as MRSA and salmonella, indicating that wild fungal peptides could have potential therapeutic applications [104]. *Russula gnathangensis* demonstrated strong antioxidant activities, suggesting its potential nutritional value for local communities [209].

#### 2.2.11. *Russula griseocarnosa*

Extracts from *R. griseocarnosa* fruit bodies have been shown to reduce oxidative damage caused by formaldehyde inhalation in mice. These bioactive-rich extracts are also used in dietary supplements and cosmetic products for their antioxidant, immune-enhancing, and anti-aging effects, supporting both overall health and skin care [210].

Extracts from *R. griseocarnosa* have been found to increase glutathione and superoxide dismutase levels in mouse serum, enhancing the body’s ability to adapt to physical exercise, resist fatigue onset, and accelerate fatigue recovery [211,212].

Postharvest NO figuration stimulates phenolic and flavonoid accumulation and enhances the antioxidant activities in *R. griseocarnosa*. Thus, NO fumigation might have potential applications to enhance the bioactive compounds and improve the antioxidant activities of *R. griseocarnosa* [213]. This mushroom contained very useful phytochemicals such as caffeic acid, flavonoids, ergosterol, phenolics, protocatechuic acid, and β-carotene [160]. Moreover, the major component in *R. griseocarnosa* was quercetin. Bioactive substances, together with rich nutritional composition, lead to *R. griseocarnosa* as a potential nutritive source [160]. Research has demonstrated that fresh fruit bodies of *R. griseocarnosa* stimulate the activities of phenylalanine ammonia-lyase (PAL) and chalcone synthase, resulting in increased phenolic and flavonoid accumulation when treated with nitric oxide fumigation. This process enhances the bioactive compounds and improves the antioxidant properties of the mushrooms [214].

#### 2.2.12. *Russula helios*, *Russula integra*, *Russula kivuensis*, and *Russula laurocerasi*

The anti-inflammatory effects of extracts from *Russula* species, including *R. alboareolata*, *R. medullata*, *R. virescens*, and *R. helios*, were studied. *R. alboareolata* showed the strongest anti-inflammatory activity, inhibiting nitric oxide, prostaglandin E2, and COX-2, with minimal cytotoxicity, suggesting its potential as an effective anti-inflammatory agent [190]. A methanolic extract of *R. integra* showed a cytotoxic effect on non-small cell lung cancer cells (NCI-H460) [70]. The ethanolic extracts of five wild mushrooms from Tanzania’s Southern Highlands were analyzed using gas chromatography–mass spectrometry, revealing 75 chemical compounds, including fatty acids, carotenoids, alkaloids, phenols, terpenes, steroids, and amino acids. Key species studied included *R. cellulata*, *R. kivuensis*, *Lactarius densifolius*, *L. gymnocarpoides*, and *Lactarius* sp. [108]. The antioxidant properties of a phenolic extract from *R. laurocerasi* were evaluated using various in vitro assays. The extract showed strong antioxidant activity, particularly in hydroxyl radical scavenging, with a low EC50 value of 0.03 mg/mL. The antioxidant effects were correlated with the presence of total phenols and flavonoids, suggesting that these polyphenols are partly responsible for the observed activity. The findings indicate that *R. laurocerasi* could be a promising source of therapeutic antioxidants [108].

#### 2.2.13. *Russula lepida*

*Russula lepida* and *P. ostreatus* extracts from Himachal Pradesh, India were screened for phytochemicals and tested for antibacterial activity. Rich in bioactive compounds, the methanol extract was most effective, particularly against *B. subtilis*, highlighting its potential as a source of new antimicrobial agents and validating their traditional medicinal uses [114]. Thermostable lectins with Cu^2+^-induced enhancement, and potent antiproliferative and antitumor activities were isolated from *R. lepida* [115]. The *R. lepida* lectins have high antitumor activity, and therefore, they can be developed into agents for cancer therapy [117]. *Russula lepida* exhibited antiproliferative activity to hepatoma Hep G2 cells and human breast cancer MCF-7 cells [115].

#### 2.2.14. *Russula luteotacta*, *Russula mairei* (=*R. nobilis*), and *Russula medullata*

In a study, it was found that catechin was highest in *R. luteotacta* (2.09 mg/g dry weight) [215]. Ethanolic extracts prepared from *R. mairei* (=*R. nobilis*, https://www.indexfungorum.org/Names/Names.asp, accessed on 26 March 2025) have been shown selective anti-inflammatory activity by decreasing the production of NO and IL-6 but not TNF-α in LPS-stimulated RAW264.7 cells [216]. The anti-inflammatory effects of extracts from *Russula* species, including *R. alboareolata*, *R. medullata*, *R. virescens*, and *R. helios*, were studied. *Russula alboareolata* showed the strongest anti-inflammatory activity, inhibiting nitric oxide, prostaglandin E2, and COX-2, with minimal cytotoxicity, suggesting its potential as an effective anti-inflammatory agent [190].

#### 2.2.15. *Russula mustelina*

*Russula mustelina*, *R. delica*, and *R. lepida* were studied at three maturity stages—immature, mature, and post-mature, and it was revealed that protein, ash, crude fibers, lipids, and energy values increased with maturity, while carbohydrates were highest at the immature stage. Minerals like potassium, phosphorus, and calcium were most abundant at the mature stage. In addition, all essential amino acids were present at the immature stage, indicating that these mushrooms are valuable nutritional resources, especially at the mature and post-mature stages [116].

#### 2.2.16. *Russula nigricans*, *Russula nobilis*, *Russula ochroleuca*, *Russula ochrocephala*, and *Russula paludosa*

Nigricanin, which has interesting biological activities, has been isolated from the fruiting bodies of *R. nigricans*. This is the first ellagic acid derivative isolated from higher fungi [182]. Nigricanin P-hydroxybenzoic and cinnamic acids were identified in ethanolic extracts of *R. nigricans*, which showed antioxidant activity through the inhibition of thiobarbituric acid reactive substances formation and oxidative hemolysis [70]. The spirodioxolactone ochroleucin A1 is responsible for the red color produced when the stalk base of *R. ochroleuca* and *R. viscida* is treated with aqueous KOH, and it easily rearranges into the isomeric dilactone ochroleucin A2. Ochroleucin A1, along with the related hemiacetal ochroleucin B (5), is derived from the oxidative condensation of monomeric units, with their structures confirmed via MS, NMR, and quantum chemical calculations of CD spectra [117]. Water extract of *R. paludosa* showed an inhibitory effect on HIV-1 reverse transcriptase [119].

#### 2.2.17. *Russula pseudocyanoxantha*

*Russula pseudocyanoxantha* was found to be rich in phenolics, flavonoids, and antioxidants, with significant antibacterial properties, particularly against Gram-positive bacteria. The ethanol extract also exhibited a notable antiproliferative effect on Hep3B cells, suggesting its potential involvement in mitochondria-mediated pathways [142].

*Russula pseudocyanoxantha* polysaccharide fraction was extracted using hot water, and the solid remnants of the extraction process, which still contained therapeutic biopolymers, were valorized. These remnants were treated with cold alkali, yielding a high-yield fraction (RP-CAP). Chemical analysis revealed the presence of various monomers, primarily β-glucan, with a homogeneous polymer of approximately 129.28 kDa. RP-CAP demonstrated potent antioxidant and immune-boosting activities, particularly at 100 μg/mL, through the TLR/NF-κB pathway. These findings suggest that RP-CAP has significant potential as a health-enhancing component in functional foods and pharmaceuticals [120].

#### 2.2.18. *Russula rosea* and *Russula rosacea*

A novel lectin with potent in vitro anti-tumor activity was isolated from *Russula rosea*, the first lectin reported from *Russula* [115]. *Russula rosacea* showed significant antitumor effects on sarcoma 180 in mice, likely due to its immunomodulating properties. Extracts from the mushroom, including those soluble in saline, hot water, and methanol, enhanced immune responses and prolonged survival in treated mice without showing cytotoxicity against cancer cell lines [217]. The fruiting bodies of *R. rosacea* were extracted using methanol and hot water. The extracts showed strong DPPH radical scavenging and chelating effects but a lower reducing power compared to BHT. With seven identified phenolic compounds, the extracts also demonstrated a moderate inhibition of acetylcholinesterase and butyrylcholinesterase and significant nitric oxide (NO) inhibition in LPS-induced cells, indicating their potential as natural sources of antioxidants and anti-inflammatory agents [218].

#### 2.2.19. *Russula senecis*

*Russula senecis*, a historically valued but neglected myco-resource, was explored for its health benefits. The ethanolic extract, rich in phenolics, flavonoids, ascorbic acid, and carotenoids, demonstrated strong antioxidant and antibiotic properties. It also showed selective anti-cancer activity against Hep3B cells, inducing apoptosis through the mitochondrial pathway. The study suggests that *R. senecis* has potential applications in the medicinal, pharmaceutical, and food industries [125]. The entire fruit bodies of *R. senecis* were used to isolate a β-glucan-enriched polysaccharide fraction (RuseHap) through consecutive hot water and cold alkaline extraction, reducing waste and fully utilizing the mushroom. The isolated RuseHap demonstrated strong antioxidant and immune-boosting properties, with potential pharmacotherapeutic applications, likely due to its interaction with Toll-like receptors (TLR2 and TLR4), leading to enhanced immune response and gene expression related to inflammation [126].

#### 2.2.20. *Russula subnigricans*

Five new chlorinated phenyl ethers, Russuphelins B, C, D, E, and F, have been isolated from *R. subnigricans*, and Russuphelins B, C and D exhibited cytotoxic activity in vitro against P388 leukemia cells [219]. A new cytotoxic substance, designated as russuphelin A, has been isolated from the mushroom *R. subnigricans* [220].

#### 2.2.21. *Russula vesca*

From *R. vesca*, two compounds, triyne acid, and triyinol, were isolated [129].

*Russula* vesca was noted for its high carbohydrate content (71%) and significant magnesium levels (14 g/kg). This mushroom also exhibited lower moisture, lipid, sodium, and phosphorus contents compared to other species analyzed [221].

*Russula delica* and *R. vesca* were among the 18 Portuguese wild mushrooms evaluated for their antioxidant properties. The study measured their radical-scavenging capacity, reducing power, and inhibition of lipid peroxidation, revealing that these mushrooms contain significant levels of antioxidants, including phenols and tocopherols. The research highlighted their potential for nutraceutical applications and emphasized the importance of managing and conserving these mushroom species [128]. Phenols, flavonoids, and antioxidant activity were assessed in aqueous and ethanolic extracts from *Agaricus macrosporus* and *R. vesca*. Aqueous extracts demonstrated higher antioxidant activity and phenol content than ethanolic extracts, with *A. macrosporus* showing more phenols and *R. vesca* exhibiting higher flavonoids. These results suggest that aqueous mushroom extracts could serve as effective substitutes for synthetic antioxidants in different industries [222].

The antibacterial potential of aqueous and ethanolic extracts from *A. macrosporus* and *R. vesca* was investigated. *Russula vesca* extracts had higher total carbohydrate and protein content. Aqueous extracts showed superior antibacterial activity compared to ethanolic ones. Notably, the aqueous extract of *R. vesca* was more effective against *Bacillus cereus* (13.6 mm), *Enterococcus faecalis* (12.1 mm), *E. coli* (16.7 mm), and *Pseudomonas aeruginosa* (10.5 mm) than gentamicin or neomycin. These results highlight the potential of mushroom extracts for diverse industrial applications [130].

#### 2.2.22. *Russula vinosa* and *Russula violeipes*

Water-extracted polysaccharides from *R. vinosa* (WRP) were separated into three fractions: WRP-1, WRP-2, and WRP-3. WRP-1, a branched β-(1→3)-glucan with a rigid helical conformation, exhibited the strongest immunostimulatory activity. In contrast, WRP-2 and WRP-3, composed of galactoglucans with more flexible structures, showed lower immunostimulatory effects. All fractions promoted macrophage proliferation, phagocytosis, and the release of nitric oxide and cytokines, indicating their potential as natural immunostimulators in the food and pharmaceutical industries [223]. Extracts from *R. densifolia*, *R. violeipes*, and *R. cyanoxantha* showed strong antioxidant activities, including ABTS and DPPH radical scavenging, along with α-glucosidase and α-amylase inhibition. They also exhibited anti-inflammatory effects by inhibiting albumin denaturation and moderate antimicrobial activity. The ethanol extract of *R. violeipes* had notable cytotoxicity with an IC50 of 56.66 mg/mL, and the extracts contained significant levels of phenols and flavonoids [137].

#### 2.2.23. *Russula virescens* and *Russula viscida*

The chemical compositions of *R. virescens* were assessed among several wild edible mushroom species from Bukovina, Romania. The study measured water, crude protein, lipids, carbohydrates, and ash content. *R. virescens* had a carbohydrate content lower than *Agaricus albolutescens*, *Boletus edulis*, and *Armillaria mellea*. Its protein content varied between 10.12% and 15.15% dry weight. Notably, *Russula* virescens exhibited higher antiradical activity compared to other species, suggesting its potential benefits in antioxidant protection [224].

*Russula virescens* exhibited an anti-inflammatory effect in the RAW 264.7 cell by suppressing the expression of STATs, a reduction in TNF-α, and NO production [225].

A Chinese study suggests that *R. virescens* has beneficial effects on blood lipid regulation. Rats given high (600 mg/kg/day) and low (300 mg/kg/day) doses of *R. virescens* via stomach perfusion for 30 days had significantly (*p* < 0.05) lower levels of total cholesterol, total low-density lipoprotein cholesterol, and triglycerides than in the hyperlipidemia control group [226]. The Chinese study above also showed that rats given high and low doses of the mushroom had lower levels of serum and liver malondialdehyde (a biomarker used to measure levels of oxidative stress), and increased levels of the enzyme superoxide dismutase [226].

The spirodioxolactone ochroleucin A1 is responsible for the red color produced when the stalk base of *R. ochroleuca* and *R. viscida* is treated with aqueous KOH, and it easily rearranges into the isomeric dilactone ochroleucin A2. Ochroleucin A1, along with the related hemiacetal ochroleucin B (5), is derived from the oxidative condensation of monomeric units, with their structures confirmed via MS, NMR, and quantum chemical calculations of CD spectra [117].

#### 2.2.24. *Russula xerampelina*

*Russula xerampelina* demonstrated antibacterial activity against *Plasmodium falciparum* [138]. The antibacterial potential of ethanolic extracts from *R. xerampelina* and *Suillus granulatus* mushrooms against *P. aeruginosa* was investigated, revealing an additive effect when combined. The extracts also displayed allelopathic effects, reducing the germination rates of *Lactuca sativa* (lettuce) and *Solanum lycopersicum* (tomato) seeds at higher concentrations. However, the seeds exhibited a positive response when analyzed for the allelopathic index, suggesting that these extracts could be effective in controlling *Pseudomonas* phytopathogens without causing significant phytotoxicity [139].

*Russula xerampelina*, known as the “shrimp mushroom”, emits a strong shellfish-like odor. Analysis using SPME and GC–MS identified trimethylamine and trimethylamine N-oxide as the primary volatile compounds, with trimethylamine responsible for its fishy, cooked-seafood aroma [140]. Table 4 lists the bioactive properties of various *Russula* sp.

## 3. Biotechnological Applications

The trace element levels in *Russula* species from the East Black Sea region were analyzed. *Russula foetens* had the lowest Hg level at 0.06 mg/kg, and *R. cyanoxantha* had the highest Cd level at 3.16 mg/kg. The study highlighted the metal bioaccumulation in these mushrooms, with specific focus on Cd and Hg levels [227]. *Russula cyanoxantha* from Dambovita County, Romania, showed iron concentrations four times higher than the average of the studied species. This indicates *R. cyanoxantha’s* strong ability to bioaccumulate iron while maintaining normal growth [228]. The antifungal effects of *R. cyanoxantha* were investigated against the plant pathogens *Fusarium moniliforme* and *F. culmorum*, which cause paleness sickness and root corrosion. Dried mushroom extracts, prepared using acetone and chloroform, exhibited significant antagonistic effects against both *Fusarium* species, with clear zones of inhibition observed, comparable to commercial antibiotics like amoxicillin and erythromycin [229]. Bacteria can colonize a wide variety of medical devices, leading to local and systemic infectious complications such as site infections, catheter-related bloodstream infections, and endocarditis [230]. Those bacteria are able to grow and adhere to almost every surface, forming architecturally complex communities termed biofilms [231]. *Russula delica* extract inhibits the biofilm production of *E. coli*, *Proteus mirabilis*, *P. aeruginosa*, and *Acinetobacter baumannii* [232]. Mercury (Hg) contamination in *Russula ochroleuca* was studied at ten unpolluted sites in northern Poland. Hg levels were 0.017 to 0.43 μg/g in caps and 0.011 to 0.24 μg/g in stipes. Caps had higher Hg concentrations than stipes, with bioconcentration factors of 0.57 to 5.6 for caps and 0.50 to 3.3 for stipes. Higher Hg levels were found in mushrooms from Trójmiejski Landscape Park. The study suggests *Russula ochroleuca* could be used as a bioindicator for environmental Hg pollution [118].

*Russula* species such as *R. atropurpurea* [233,234,235], *R. bresadolae* [236,237], *R. ochroleuca* [237], and *R. pumila* [238] can accumulate remarkably high concentrations of Zn and substantially contribute to the cycling and environmental sequestration of metal elements. *Russula* species can accumulate and translocate heavy metals under natural pH conditions. While the concentrations of iron (Fe), zinc (Zn), and copper (Cu) varied by species, these mushrooms exhibit a low capacity to accumulate these metals but demonstrate significant mobility within their fruiting bodies [239]. The production of vinegar from the wild edible mushroom *R. delica* using microwave-assisted enzymatic hydrolysis extraction resulted in a product with high nutritional value, significant antioxidant activity, and a unique aroma. The process yielded a vinegar with 10.95% alcohol content, 5.60% acetic acid, and notable levels of phenolic compounds. Thirteen volatile compounds were identified, contributing to its distinct aroma. This study represents the first analysis of vinegar derived from a mushroom, showcasing its potential for future commercial production [240].

*Russula vinosa* Lindblad, a traditional food and medicinal resource rich in polyphenolic compounds, was mixed fermented with *Saccharomyces boulardii* and *Lactobacillus lactis*. The ethanol extract of the mixed bacterial fermentation product (EMFP) exhibited enhanced antioxidant activity and introduced 186 new compounds, including organic and phenolic acids. EMFP addition significantly improved bread quality by reducing hardness and chewiness while enhancing resilience and antioxidant properties. However, excessive EMFP negatively affected sensory attributes. A 0.5% EMFP addition was optimal for balancing quality, sensory evaluation, and antioxidant benefits, supporting its potential application in functional foods [241].

A novel laccase from *R. virescens* was purified using chromatography and gel filtration. The 69 kDa monomeric enzyme has an N-terminal sequence AIGPTAELVV and optimal activity at pH 2.2 and 60 °C. It was inhibited by Cu^2+^ and other inhibitors. The laccase degrades phenolic compounds and decolorizes various laboratory and textile dyes, with a Km of 0.1 mM for specific substrates [242]. Halogen speciation analysis using HPLC-ICPMS/MS revealed the presence of dichloroacetic acid (DCAA) in *R. nigricans* at concentrations of 23–37 mg/kg. This compound was not detected in other *Russula* species or mushrooms from the same regions, suggesting that *R. nigricans* may biosynthesize DCAA. This finding challenges the traditional view of DCAA as merely a pollutant from water disinfection, highlighting its natural occurrence in living organisms [243]. Significant bioaccumulation of manganese and nickel of *R. delica* underscores the importance of understanding heavy metal accumulation. This knowledge is crucial for assessing potential health risks and ecological impacts, particularly in regions with varying soil metal concentrations. Monitoring and analyzing these metal levels can inform safer foraging practices and contribute to environmental health assessments [244]. *Russula delica* mushroom/bentonite clay (RDBNC) was tested as a low-cost bionanosorbent for removing methylene blue (MB) and malachite green (MG) dyes. Adsorption followed Freundlich isotherm and pseudo-second-order kinetics, driven by π–π interactions, hydrogen bonding, and electrostatic forces. RDBNC maintained efficiency after four cycles, and thermodynamic analysis confirmed spontaneous, exothermic adsorption. It also exhibited antibacterial effects against E. coli, making it a promising eco-friendly material for dye removal [245]. Table 5 presents the trace element levels and biotechnological applications of various *Russula* species.

## 4. Toxicity

*Russula subnigricans* is a toxic mushroom known for causing fatal poisoning when mistakenly ingested. The identification of its responsible toxin remained elusive for about 50 years due to the toxin’s instability and frequent misidentification of the mushroom. Recently, researchers isolated the unstable toxin and identified its structure. In addition, they discovered a unique chemical marker, cyclopropylacetyl-(R)-carnitine, which helps distinguish *R. subnigricans* from similar species [246]. A case involving seven family members poisoned by *R. subnigricans* revealed symptoms ranging from gastrointestinal issues to rhabdomyolysis, with one fatality. This case highlights that *R. subnigricans* can cause rhabdomyolysis and emphasizes the need for early recognition and intensive supportive care for affected individuals [247]. Rhabdomyolytic mushroom poisoning, a newly recognized syndrome, is on the rise globally, with *R. subnigricans* identified as a cause. This report details the first recorded case in Korea, involving a 51-year-old man who developed rhabdomyolysis, acute kidney injury, and severe complications, ultimately leading to death. The case underscores the importance of considering mushroom poisoning, particularly from *R. subnigricans*, in unexplained rhabdomyolysis cases [248]. *Russula subnigricans* poisoning, a newly identified syndrome, causes severe rhabdomyolysis, acute kidney injury, and cardiomyopathy. In a recent case series, two out of six patients died from severe complications, including metabolic acidosis and irreversible shock. This poisoning should be considered in cases of rhabdomyolysis with unknown origins [145].

The essential and non-essential element contents of eight *Russula* species were evaluated, revealing high levels of potassium, magnesium, and calcium. Elevated metal concentrations were found in *R. risigallina*, *R. olivacea*, and *R. velenovskyi*, with chromium levels in *R. risigallina* and *R. olivacea* exceeding safe limits. Health risk indices suggested potential risks from chromium and cadmium in some species [147]. The potential myotoxicity of various edible mushrooms, including *Russula* spp., *Cantharellus cibarius*, *Albatrellus ovinus*, and *Leccinum versipelle*, was assessed after reports of *Tricholoma flavovirens* causing delayed rhabdomyolysis and fatalities. In a study involving 86 mice, the consumption of these mushrooms at high doses led to increased plasma creatine kinase activity, indicating potential muscle damage, though no histological changes were observed in muscle or liver tissues [249]. Myotoxic mushroom poisoning was studied in Thailand over a 5-year period, involving 41 patients. Symptoms included gastrointestinal issues and myalgia, with rhabdomyolysis developing 24–48 h after ingestion. Some mushrooms were identified as *Russula* species. Key issues were acute kidney injury (51.5%), hyperkalemia (33.3%), and a 26.8% mortality rate. Effective treatment required early detection and monitoring of serum potassium, creatinine, and CPK levels, with interventions including fluids, urine alkalinization, and dialysis [127]. A case series of five patients revealed a unique instance of hemolysis in a patient with glucose-6-phosphate dehydrogenase deficiency, suggesting that the mushroom’s toxin may trigger hemolysis in susceptible individuals. This finding underscores the need for further research on the toxic effects of *R. subnigricans* [250].

Mushroom poisoning cases, including *R. subnigricans*, are on the rise, with increasing severity and fatality. A report described a family with *R. subnigricans* poisoning complicated by severe rhabdomyolysis. A 64-year-old man initially misdiagnosed with myocardial infarction, later found to have rhabdomyolysis from mushroom poisoning, was hospitalized alongside two other family members with similar symptoms. After intensive care and fluid resuscitation, all patients recovered without complications. Early identification and supportive care are crucial in managing mushroom poisoning cases [251]. Even 36 years after the Chernobyl disaster, consuming wild mushrooms in Ukraine’s Polissya remains risky due to high radionuclide contamination. *Imleria badia* and *Tricholoma equestre* showed the highest 137Cs levels, while *R. emetica* had the highest 90Sr levels. Annual effective doses from consuming these mushrooms ranged from 0.0014 to 8.71 mSv, depending on contamination levels [252]. Cadmium (Cd) and lead (Pb) levels in wild mushrooms from Poland’s “Green Lungs” region were assessed and compared to those in cultivated species. *R. vinosa* exhibited the highest Pb level among wild mushrooms at 2.61 μg/g, while *R. heterophylla* had a lower Cd concentration at 0.10 μg/g. Cultivated mushrooms generally showed lower levels of both metals. While Pb intake from wild mushrooms is considered safe, consuming *Rozites caperatus* and *Boletus chrysenteron* may exceed the provisional tolerable monthly intake (PTMI) for Cd, posing a potential risk of toxicity [253]. *Russula virescens* can accumulate heavy metals, posing health risks, with Cd, Pb, Cu, Zn, Co, Cr, Mn, Ni, and Fe analyzed in mushrooms and soil using the DGT technique. A correlation between R-values (metal resupply capacity) and bioaccumulation factors (BAFs) showed that faster resupply increases metal uptake. While soil contamination was below legal limits, high Cu levels in mushrooms may pose risks, especially for children [254].

## 5. Cultivation Challenges of *Russula*: Current Knowledge and Limitations

The cultivation of *Russula* presents significant challenges due to its obligate ectomycorrhizal nature, requiring symbiotic relationships with specific host trees for successful growth and fruiting [157,255,256,257]. Unlike commercially cultivated saprotrophic mushrooms such as *Agaricus bisporus* or *P. ostreatus*, *Russula* species, including *R. brevipes* and *R. griseocarnosa,* depend entirely on living root systems of host trees for nutrient exchange and fruiting body formation [65,258,259,260]. This biological constraint has hindered artificial cultivation efforts, as these mushrooms typically fail to produce fruiting bodies in axenic culture or on standard artificial substrates [256,261,262].

Limited success has been achieved through mycorrhizal synthesis approaches with particular *Russula* species [263]. For example, researchers have demonstrated the formation of functional ectomycorrhizae between *R. brevipes* and *Pinus densiflora* seedlings under controlled laboratory conditions, though reliable fruiting body production remains elusive [264]. Nevertheless, *R. griseocarnosa*, cannot be cultivated artificially and is solely harvested from natural habitats. Currently, there is limited understanding of controlled *Russula* cultivation and its associated microbial interactions [39,157]. Another *Russula* sp., has shown some promise in laboratory mycelial growth experiments but similarly fails to fruit without its natural tree partners (Wang & Guerin-Laguette, unpublished).

The cultivation barriers for *Russula* species are multifaceted. Each species exhibits specific host requirements, with *R. cyanoxantha* preferentially associating with *Fagus* trees in European forests, while *R. emetica* shows a stronger affinity for *Betula* species in northern ecosystems [16,261]. In addition, *Russula* mycelium grows significantly slower in culture compared to commercial mushroom species, and its complex nutrient requirements are difficult to replicate in artificial systems [265,266]. A patent proposes an innovative method for cultivating *Russula* species using sunflower byproducts as a substrate. The formula combines sunflower plates, stalks, seed shells, and cakes with bran, lime, and gypsum (63–65% moisture), reportedly achieving robust mycelial growth and impressive 130–142% biological efficiency. This approach not only offers a potential cultivation method but also provides sustainable utilization of agricultural waste material [267]. The fundamental biological understanding of the molecular dialogue between *Russula* fungi and their host plants remains incomplete, further complicating efforts to cultivate these fungi.

Current production, therefore, relies heavily on wild harvesting, raising concerns about sustainability and ecological impacts. Some researchers have proposed “wild-simulated” cultivation methods, where forest areas are intentionally inoculated with desired *Russula* species, as demonstrated with *R. olivacea* in managed oak forests [268,269,270]. While not actual cultivation in the agricultural sense, this approach may help conserve natural populations while allowing for some level of production management. As research continues, particularly in understanding the molecular basis of the *Russula*–host interaction, opportunities may arise to develop more reliable cultivation methods for these ecologically and gastronomically important fungi.

## 6. Future Works and Drawbacks

Future research on *Russula* should focus on several key areas to fully unlock its medicinal potential. One important direction is the expanded phytochemical profiling of *Russula* species, using advanced analytical techniques like metabolomics and high-throughput screening. This would help comprehensively identify and characterize novel bioactive compounds with therapeutic potential. Special attention should be given to compounds whose production may depend on symbiotic interactions with host trees. In addition, there is a need for rigorous clinical trials and pharmacological studies to validate the efficacy and safety of these compounds in treating specific diseases. While in vitro and animal studies provide valuable insights, translating these findings into human health benefits requires well-designed clinical research.

Biotechnological applications also hold great promise for *Russula*. Future work should explore innovative approaches to cultivate these mushrooms sustainably, optimize yield, particularly by developing cultivation systems that maintain essential ectomycorrhizal relationships and develop efficient methods for extracting bioactive compounds. Advancing genomic and molecular studies on *Russula* could provide deeper insights into the biosynthetic pathways and host–symbiont signaling mechanisms responsible for producing these valuable compounds, potentially enabling the genetic engineering of *Russula* strains with enhanced therapeutic properties or facultative growth capabilities. Safety and toxicology research remains a critical area of focus. Although many *Russula* species are safe for consumption, the potential toxicity of some species necessitates comprehensive studies to establish safe consumption levels and identify any harmful effects. This is particularly important, as *Russula* species are increasingly considered for integration into modern medicinal practices.

However, there are several drawbacks that must be addressed. One significant challenge is the limited clinical evidence supporting the therapeutic efficacy of *Russula* bioactive compounds in humans. While existing studies offer promising results, most have been conducted in vitro or on animal models, which may not fully translate to human applications. The current inability to cultivate many *Russula* species without their natural host trees severely limits the standardized production of bioactive compounds for clinical research. In addition, the concentration of bioactive compounds in *Russula* species can vary widely, depending on factors such as geographical location, environmental conditions, host tree species, and cultivation practices. This variability complicates the standardization necessary for therapeutic use. Another concern is the potential toxicity of some *Russula* species. Despite their widespread use in traditional medicine and as food, not all species have been thoroughly studied for safety, which could pose health risks. The challenges of large-scale cultivation also present obstacles, as *Russula* mushrooms require specific growth conditions that are difficult to replicate artificially, and they are prone to contamination, potentially limiting the availability of *Russula*-derived products. Finally, integrating *Russula* bioactive compounds into pharmaceuticals or nutraceuticals may face regulatory hurdles, particularly for compounds derived from symbiotic cultivation systems, as obtaining approval from health authorities often requires extensive safety and efficacy data. These regulatory challenges could slow down the development and commercialization of *Russula* products despite their potential benefits. Addressing these drawbacks will be essential to realizing the full therapeutic potential of *Russula* in the future.

## 7. Conclusions

The current understanding of *Russula* species highlights their considerable pharmacological potential, supported by the presence of bioactive compounds with antioxidant, anti-inflammatory, antimicrobial, and immunomodulatory properties. However, translating this potential into practical applications requires addressing several critical research challenges.

A primary need lies in achieving comprehensive taxonomic clarification through integrated approaches that combine morphological and molecular techniques. This will help resolve existing ambiguities related to species complexes and cryptic diversity. In addition, detailed studies are necessary to elucidate the mechanisms of bioactive compounds, including their molecular targets, pharmacokinetics, and pharmacodynamics. Rigorous toxicological assessments must also be conducted to establish safety profiles for potential medicinal use.

Furthermore, the development of standardized protocols for cultivation and extraction is essential to ensure consistent and reproducible yields of bioactive compounds. Addressing these gaps through multidisciplinary research will not only validate the *Russula* species as promising candidates for pharmaceutical development but also enhance our broader understanding of fungal secondary metabolism and its ecological roles. Moving forward, prioritizing translational research will be key to bridging ethnomycological knowledge with clinically validated therapeutic applications.

## Figures and Tables

**Table 1:** Edibility status of *Russula* species documented in this review.

*Russula* Species	Edibility	References
*Russula acrifolia*	Not edible	[80]
*R. adusta*	Edible (caution)	[81]
*R. aeruginea*	Edible	[82]
*R. alboareolata*	Edible	[83,84,85]
*R. alatoreticula*	Unknown	[86,87]
*R. albonigra*	Not edible	[81,88,89]
*R. alnetorum*	Edible (caution)	[90]
*R. alutacea*	Edible	[83,84,85]
*R. amarissima*	Not edible	[91]
*R. aurea*	Edible	[92,93,94,95]
*R. aurora*	Unknown	[96]
*R. brevipes*	Edible (caution)	[97]
*R. chloroides*	Edible (caution)	[98]
*R. cyanoxantha*	Edible	[80,99]
*R. delica*	Edible (caution)	[80,99]
*R. densifolia*	Not edible	[100]
*R. emetica*	Poisonous	[101]
*R. flavobrunnea* var. *aurantioflava*	Unknown	[102]
*R. foetens*	Not edible	[103]
*R. fragilis*	Not edible	[104]
*R. fragrantissima*	Not edible	[100]
*R. griseocarnosa*	Edible (caution)	[105]
*R. helios*	Edible	[83,84,85]
*R. integra*	Edible	[70]
*R. japonica*	Edible (caution)	[106,107]
*R. kivuensis*	Unknown	[108]
*R. laurocerasi*	Unknown	[109]
*R. lepida*	Edible	[91,110,111,112,113,114,115]
*R. luteotacta*	Edible (caution)	[80]
*R. mairei (=R. nobilis)*	Edible (caution)	[80,99]
*R. medullata*	Edible	[83,84,85]
*R. monspeliensis*	Edible	[83,84,85]
*R. mustelina*	Unknown	[113,116]
*R. nigricans*	Poisonous	[101]
*R. nobilis*	Edible (caution)	[80,99]
*R. ochroleuca*	Edible (caution)	[117,118]
*R. ochrocephala*	Unknown	[102]
*R. paludosa*	Edible	[119]
*R. pseudocyanoxantha*	Edible (caution)	[86,120,121]
*R. queletii*	Not edible	[122]
*R. rosacea*	Not edible	[100]
*R. sanguinaria*	Not edible	[123]
*R. senecis*	Not edible	[124,125,126]
*R. subnigricans*	Poisonous	[127]
*R. vesca*	Edible	[128,129,130]
*R. vinosa*	Edible (caution)	[131,132,133,134,135,136]
*R. violeipes*	Edible (caution)	[137]
*R. virescens*	Edible	[83,84,85]
*R. viscida*	Not edible	[117]
*R. xerampelina*	Edible	[138,139,140]

**Table 2:** Polysaccharides from various *Russula* species and their associated biological activities.

Species	Polysaccharide	Biological Activity	Reference
*R. adusta*	RAP (5763 Da)	Antioxidant activity, scavenges hydroxyl radicals	[167]
*R. alatoreticula*	Rusalan, RualaCap, RualaHap	Antioxidant, antibacterial (*S. aureus*, *B. subtilis*), immune-stimulatory, anticancer (Hep3B)	[86,87,105]
*R. albonigra*	β-glucan, heteroglycan	Antioxidant, macrophage activation, NO production, splenocyte and thymocyte proliferation	[81,88,89]
*R. alutacea*	Acetylated and sulfated polysaccharides	Antioxidant, anti-inflammatory, immune-modulatory	[85,151,152]
*R. aurea*	Water-soluble polysaccharides	Antitumor (sarcoma 180, Ehrlich solid cancer), antioxidant, mutagenic and antimutagenic potential	[92,93,94]
*R. delica*	Water-soluble polysaccharides	Anti-leishmanial (inhibits *L. donovani* amastigotes)	[81]
*R. emetica*	Bioactive polysaccharides	Antidiabetic, antihypertensive	[170]
*R. griseocarnosa*	PRG, PRG1-1, RGP1, RGP2	Antioxidant, immunomodulatory (macrophage activation, NF-κB, MAPK pathways), anticancer (HeLa, SiHa), hematopoietic function improvement	[153,154,155,156,157,158,159,160,161,163]
*R. pseudocyanoxantha*	RP-CAP, RP-HAP	Antioxidant, immune-boosting (macrophage proliferation, TLR/NF-κB pathway)	[120,121]
*R. senecis*	Rusenan	Antioxidant (free radical scavenging, Fe^2^⁺ chelation), immune-stimulatory (macrophage activation, NO and ROS production, gene transcription)	[124]
*R. virescens*	RVP, RVP-1, RVP-2, SRVP1	Antidiabetic, anticancer, antioxidant, immunological, anticoagulant, antibacterial	[85,92,95,164,165]
*R. vinosa*	RP-1, RP-5, CA-S, CA-L	Antioxidant, anticancer (HeLa, HepG2), immunomodulatory (macrophage activation, NF-κB pathway), anti-inflammatory (ulcerative colitis)	[131,132,133,134,135,136,137]

**Table 3:** Summary of triterpenoids isolated from various *Russula* species and their bioactivities.

Species	Compound Type	Specific Compounds	Biological Activity	Reference
*Russula amarissima*	Aristolane sesquiterpenes, seco-cucurbitane triterpene	Four aristolane sesquiterpenes, seco-cucurbitane triterpene (3,4-secocucurbita-4, 24E-diene-3-hydroxy-26-carboxylic acid)	-	[91]
*R. brevipes*	Triterpenoids	Lactarorufin A, Russulactarorufin	-	[97]
*R. cyanoxantha*	Phytosphingosine-derived ceramides	Russulamide and other ceramides	-	[172]
*R. delica*	Protoilludane-type sesquiterpenoids	New norsesquiterpenoid (russulanorol), eight known sesquiterpenoids: lactarorufin A, blennin C, furandiol, lactarorufin B, lactarolide A, 14-hydroxylactarolide A, 3-O-methyllactarolide B, isolactarorufin	-	[174,175]
*R. foetens*	Marasmane sesquiterpenes, ergosterol	New marasmane sesquiterpene (russulfoen), known marasmane sesquiterpenes: 7α,8α,13-trihydroxy-marasm-5-oic acid γ-lactone, 8α,13-dihydroxy-marasm-5-oic acid γ-lactone, ergosterol, (1R,2R)-1-phenylpropane-1,2-diol	Poisonous with gastrointestinal irritants	[176,177,187]
*R. japonica*	Illudoid sesquiterpenes	Russujaponols A–F, neurite outgrowth-promoting sesquiterpenes	Neurite outgrowth-promoting activity; potential anticancer properties	[106,107]
*R. lepida*	Cucurbitane triterpenes, Aristolane sesquiterpenes	Cucurbitane-type triterpenoids, lepidolide, rulepidadiol, rulepidatriol, rulepidol, lepidamine	Potential type-2 diabetes and obesity treatment through PTP1B inhibition	[110,111,112]
	Seco-cucurbitane triterpenes	(24E)-3,4-seco-cucurbita-4,24-diene-3,26,29-trioic acid, rulepidadiol, rulepidatriol	PTP1B and T-cell PTP activity inhibition for potential diabetes and obesity treatment	
*R. nobilis*	Sesquiterpenoids	Velutinal esters, Russulanobilines A-C	Chemical defense against predators, parasites, and microorganisms	[183]
*R. queletii*	Bioactive compounds	Piperalol, piperdial	Antimicrobial and anticancer properties	[122]
*R. rosacea*	Triterpenes	Rosacea acids A and B	-	[184]
*R. sanguinaria*	Lactarane-type sesquiterpenoids	Sangusulactones A-C, blennin A, 15-hydroxyblennin A	Anti-inflammatory potential	[123]
*R. vinosa*	Triterpenoids, sesquiterpenoids	(1R,2S)-1-phenylpropane-1,2-diol, isolactarorufin, lactarorufin A, 8α,13-dihydroxy-marasm-5-oic acid γ-lactone, 7α,8α,13-trihydroxy-marasm-5-oic acid γ-lactone	Growth-regulating effects on plant species like lettuce	[186]
*R. virescens*	Terpenoids, fatty acids, amino acids	633 phytochemicals including polyphenols, terpenoids	Linked to cancer treatment pathways (HSP90AA1, AKT3); Nutritional and medicinal potential	[143]

**Table 4:** Bioactive properties of various *Russula* species.

Species	Key Findings	Active Compounds	References
*Russula aeruginea*	- Exhibits potent antifungal activity against *F. equiseti*. - Significant antioxidant activity confirmed through various assays. - Potential source for developing antimicrobial and antioxidant drugs.	Not specified	[82,90]
*R. alnetorum*	- No specific bioactivities mentioned in the text.	Not specified	[90]
*R. brevipes*	- Strong antibacterial activity against *B. subtilis*. - Antioxidant activity with EC50 values of 0.89 mg/mL (fruiting bodies) and 7.08 mg/mL (mycelia) in DPPH assay. - Decoction and infusion methods show superior radical scavenging and metal ion chelating activities. - Contains essential amino acids, organic acids, and nutraceuticals like betaine and carnitine.	Phenolic compounds, essential amino acids, betaine, carnitine	[193,195,196]
*R. fragrantissima*	- No specific bioactivities mentioned in the text.	Not specified	[90]
*R. nobilis*	- No specific bioactivities mentioned in the text.	Not specified	[90]
*R. ochroleuca*	- Contains spirodioxolactone ochroleucin A1 and hemiacetal ochroleucin B, responsible for color changes with KOH treatment. - Structures confirmed by MS, NMR, and quantum chemical calculations.	Ochroleucin A1, ochroleucin B	[117]
*R. alboareolata*	- Induces apoptosis in L929, HepG2, and HeLa cells. - Strong anti-inflammatory activity, inhibiting nitric oxide, prostaglandin E2, and COX-2. - Potential use in dietary supplements or chemoprevention.	Ethanolic extract (apoptosis-inducing compounds)	[188,189,190]
*R. alatoreticula*	- Contains phenols, flavonoids, ascorbic acid, β-carotene, and lycopene. - Exhibits strong antioxidant, antibacterial, and anti-cancer properties. - Effective against *B. subtilis*, *E. coli*, *K. pneumoniae*, *L. monocytogenes*, *S. aureus*, and *S. typhimurium*.	Phenols, flavonoids, ascorbic acid, β-carotene, lycopene, pyrogallol, cinnamic acid	[86,87]
*R. alveolata*	- Contains bioactive compounds like volatile oil, sterols, triterpenes, carotenoids, and saponosides. - Rich in essential amino acids (phenylalanine, valine, threonine, isoleucine, methionine, leucine, lysine).	Volatile oil, sterols, triterpenes, carotenoids, saponosides, essential amino acids	[102]
*R. aruea*	- Contains a new isolactarane sesquiterpene and 11 known compounds (sesquiterpenes, sterols, allitol, fatty acids). - Sesquiterpenes may serve as chemotaxonomic markers.	Isolactarane sesquiterpene, sterols, allitol, fatty acids	[150]
*R. aurora*	- Major phenolic compound is gallic acid. - Contains fumaric acid and catechin hydrate.	Gallic acid, fumaric acid, catechin hydrate	[96]
*R.* cf. *compressa*	- Contains bioactive compounds like volatile oil, sterols, triterpenes, carotenoids, and saponosides. - Rich in essential amino acids (phenylalanine, valine, threonine, isoleucine, methionine, leucine, lysine).	Volatile oil, sterols, triterpenes, carotenoids, saponosides, essential amino acids	[102]
*R.* cf. *foetentoides*	- Contains gallic acid as the most concentrated phenolic compound. - High antioxidant potential with significant ABTS inhibition, flavonoid, and total phenolic content.	Gallic acid, flavonoids, phenolic compounds	[103]
*R. chloroides*	- Contains phenolic acids and flavonoids like ferulic acid, gallic acid, and myricetin. - Enhances GST (glutathione S-transferase) activity.	Ferulic acid, gallic acid, myricetin	[182]
*R. cutefracta (*=*R. cyanoxantha)*	- Inhibits degranulation in mast cells. - Contains ergosta-4,6,8(14),22-tetraen-3-one (ergone), a bioactive steroid with cytotoxic and anti-proliferative activity. - Lectins show higher agglutination activity at 4 °C.	Ergosta-4,6,8(14),22-tetraen-3-one (ergone), lectins	[197,199,200]
*R. cyanoxantha*	- Antifungal activity against *M. canis* and antibacterial activity against *P. putida*. - Contains a new phytosphingosine-type ceramide and nine other compounds.	Phytosphingosine-type ceramide, ergone	[172,198]
*R. delica*	- Ethanolic extract shows antioxidant and antimicrobial activities. - Contains 8.71 ± 0.56 μg/mg flavonoids and 47.01 ± 0.29 μg/mg phenolic compounds. - Major phenolic component is catechin (5.33 mg/L). - Contains glycosaminoglycans, essential amino acids, and fatty acids (palmitic, oleic, linoleic acids). - Ethyl acetate extracts show cytotoxicity against prostate carcinoma (PC-3) cells.	Catechin, glycosaminoglycans, essential amino acids, fatty acids	[196,201,202,203,206]
*R. densifolia*	- Extracts show strong antioxidant activities (ABTS, DPPH radical scavenging). - Inhibits α-glucosidase and α-amylase. - Anti-inflammatory effects by inhibiting albumin denaturation. - Moderate antimicrobial activity.	Phenols, flavonoids	[137]
*R. emetica* (M12)	- Shows multidrug resistance (MDR) reversal activity in paclitaxel-resistant Pgp-positive cancer cells. - Enhances doxorubicin’s cytotoxicity in resistant cells.	Not specified	[207]
*R. flavobrunnea* var. *aurantioflava*	- Contains bioactive compounds like volatile oil, sterols, triterpenes, carotenoids, and saponosides. - Rich in essential amino acids (phenylalanine, valine, threonine, isoleucine, methionine, leucine, lysine).	Volatile oil, sterols, triterpenes, carotenoids, saponosides, essential amino acids	[102]
*R. foetens*	- Contains gallic acid as the most concentrated phenolic compound. - High antibacterial and antifungal activities, particularly against *Staphylococcus aureus* and *F. equiseti*. - Significant antioxidant potential with high DPPH inhibition and reducing power.	Gallic acid, flavonoids, phenolic compounds	[103]
*R. fragilis*	- Protein extracts show antimicrobial effects against common hospital pathogens. - Significant inhibition of pathogens like MRSA and *Salmonella*.	Proteins, peptides	[104]
*R. fragrantissima*	- No specific bioactivities mentioned in the text.	Not specified	[90]
*R. mgnathangensis*	- Demonstrates strong antioxidant activities. - Potential nutritional value for local communities.	Not specified	[209]
*R. griseocarnosa*	- Reduces oxidative damage caused by formaldehyde inhalation in mice. - Used in dietary supplements and cosmetics for antioxidant, immune-enhancing, and anti-aging effects. - Increases glutathione and superoxide dismutase levels in mouse serum. - Contains caffeic acid, flavonoids, ergosterol, phenolics, protocatechuic acid, and β-carotene. - Major component is quercetin.	Caffeic acid, flavonoids, ergosterol, phenolics, protocatechuic acid, β-carotene, quercetin	[163,210,211,212,213,214]
*R. helios*	- Anti-inflammatory effects by inhibiting nitric oxide, prostaglandin E2, and COX-2. - Minimal cytotoxicity.	Not specified	[190]
*R. integra*	- Methanolic extract shows cytotoxic effect on non-small cell lung cancer cells (NCI-H460).	Not specified	[70]
*R. kivuensis*	- Ethanolic extracts contain 75 chemical compounds, including fatty acids, carotenoids, alkaloids, phenols, terpenes, steroids, and amino acids.	Fatty acids, carotenoids, alkaloids, phenols, terpenes, steroids, amino acids	[108]
*R. laurocerasi*	- Phenolic extract shows strong antioxidant activity, particularly in hydroxyl radical scavenging. - Contains total phenols and flavonoids.	Phenols, flavonoids	[109]
*R. lepida*	- High levels of protein (38.08–38.52%), crude fiber (9.59–19.78%), carbohydrates (39.29–41.64%), ash (12.7–13.80%), and fat (4.06–5.70%). - Rich in potassium, phosphorus, calcium, and magnesium. - Contains 18 amino acids, with glutamic acid and valine as predominant. - Thermostable lectins with Cu^2+^-induced enhancement, potent antiproliferative and antitumor activities. - Antiproliferative activity against hepatoma Hep G2 cells and breast cancer MCF-7 cells.	Lectins, amino acids, phenols, flavonoids	[113,114,115]
*R. luteotacta*	- Contains high levels of catechin (2.09 mg/g dry weight).	Catechin	[215]
*R. mairei (=R. nobilis)*	- Ethanolic extracts show selective anti-inflammatory activity by decreasing NO and IL-6 production in LPS-stimulated RAW264.7 cells.	Not specified	[216]
*R. medullata*	- Anti-inflammatory effects by inhibiting nitric oxide, prostaglandin E2, and COX-2. - Minimal cytotoxicity.	Not specified	[190]
*R. mustelina*	- High levels of protein (38.08–38.52%), crude fiber (9.59–19.78%), carbohydrates (39.29–41.64%), ash (12.7–13.80%), and fat (4.06–5.70%). - Rich in potassium, phosphorus, calcium, and magnesium. - Contains 18 amino acids, with glutamic acid and valine as predominant. - Protein, ash, crude fibers, lipids, and energy values increase with maturity.	Amino acids, phenols, flavonoids	[113,116]
*R. nigricans*	- Contains nigricanin, the first ellagic acid derivative isolated from higher fungi. - Ethanolic extracts contain P-hydroxybenzoic and cinnamic acids, showing antioxidant activity.	Nigricanin, P-hydroxybenzoic acid, cinnamic acid	[70,182]
*R. ochrocephala*	- Contains bioactive compounds like volatile oil, sterols, triterpenes, carotenoids, and saponosides. - Rich in essential amino acids (phenylalanine, valine, threonine, isoleucine, methionine, leucine, lysine).	Volatile oil, sterols, triterpenes, carotenoids, saponosides, essential amino acids	[102]
*R. paludosa*	- Water extract shows inhibitory effect on HIV-1 reverse transcriptase.	Not specified	[119]
*R. pseudocyanoxantha*	- Rich in phenolics, flavonoids, and antioxidants. - Significant antibacterial properties, particularly against Gram-positive bacteria. - Ethanol extract shows antiproliferative effect on Hep3B cells. - Polysaccharide fraction (RP-CAP) exhibits potent antioxidant and immune-boosting activities through the TLR/NF-κB pathway.	Phenolics, flavonoids, β-glucan	[120,142]
*R. rosea*	- Contains a novel lectin with potent in vitro anti-tumor activity.	Lectin	[115]
*R. rosacea*	- Shows significant antitumor effects on sarcoma 180 in mice. - Enhances immune responses and prolongs survival in treated mice. - Methanol and hot water extracts show strong DPPH radical scavenging, chelating effects, and NO inhibition. - Contains seven identified phenolic compounds.	Phenolic compounds	[208,217,218]
*R. senecis*	- Ethanolic extract rich in phenolics, flavonoids, ascorbic acid, and carotenoids. - Demonstrates strong antioxidant, antibiotic, and selective anti-cancer activity against Hep3B cells. - β-glucan-enriched polysaccharide fraction (RuseHap) shows immune-boosting properties through TLR2 and TLR4 interaction.	Phenolics, flavonoids, ascorbic acid, carotenoids, β-glucan	[124,125]
*R. subnigricans*	- Contains five new chlorinated phenyl ethers (Russuphelins B, C, D, E, and F). - Russuphelins B, C, and D exhibit cytotoxic activity against P388 leukemia cells. - Russuphelin A is a new cytotoxic substance.	Russuphelins A-F	[219,220]
*R. vesca*	- Contains triyne acid and triyinol. - High carbohydrate content (71%) and significant magnesium levels (14 g/kg). - Aqueous and ethanolic extracts show antioxidant and antibacterial activities. - Aqueous extract is effective against *B. cereus*, *E. faecalis*, *E. coli*, and *P. aeruginosa*.	Triyne acid, triyinol, phenols, flavonoids	[129,130,222]
*R. vinosa*	- Water-extracted polysaccharides (WRP) separated into three fractions: WRP-1, WRP-2, and WRP-3. - WRP-1, a branched β-(1→3)-glucan, exhibits the strongest immunostimulatory activity. - WRP-2 and WRP-3, composed of galactoglucans, show lower immunostimulatory effects. - All fractions promote macrophage proliferation, phagocytosis, and release of nitric oxide and cytokines.	β-(1→3)-glucan, galactoglucans	[223]
*R. violeipes*	- Extracts show strong antioxidant activities (ABTS, DPPH radical scavenging). - Inhibits α-glucosidase and α-amylase. - Anti-inflammatory effects by inhibiting albumin denaturation. - Moderate antimicrobial activity. - Ethanol extract shows notable cytotoxicity with an IC50 of 56.66 mg/mL.	Phenols, flavonoids	[137]
*R. virescens*	- Carbohydrate content lower than *A. albolutescens*, *B. edulis*, and *A. mellea*. - Protein content varies between 10.12% and 15.15% dry weight. - Higher antiradical activity compared to other species. - Anti-inflammatory effect in RAW 264.7 cells by suppressing STATs, reducing TNF-α, and NO production. - Beneficial effects on blood lipid regulation in rats, reducing cholesterol, LDL, and triglycerides. - Reduces oxidative stress biomarkers (malondialdehyde) and increases superoxide dismutase levels.	Not specified	[224,225,226,227,228,229,230,231,232,233,234,235,236]
*R. viscida*	- Contains spirodioxolactone ochroleucin A1 and hemiacetal ochroleucin B, responsible for color changes with KOH treatment. - Structures confirmed by MS, NMR, and quantum chemical calculations.	Ochroleucin A1, ochroleucin B	[117]
*R. xerampelina*	- Demonstrates antibacterial activity against *P. falciparum*. - Ethanolic extracts show antibacterial potential against *P. aeruginosa*. - Exhibits allelopathic effects, reducing germination rates of *Lactuca sativa* (lettuce) and *Solanum lycopersicum* (tomato) seeds at higher concentrations. - Emits a strong shellfish-like odor due to trimethylamine and trimethylamine N-oxide.	Trimethylamine, trimethylamine N-oxide	[138,139,140]

**Table 5:** Trace element levels and biotechnological applications of various *Russula* species.

Species	Trace Elements	Findings	Reference
*Russula cyanoxantha*	Cd: 3.16 mg/kg	- Highest Cd level. - Iron concentrations four times higher than average. - Significant antifungal effects against *Fusarium* spp.	[228,229]
*R. delica*	-	- Inhibits biofilm production of *E. coli*, *P. mirabilis, P. aeruginosa,* and *A. baumannii*. - High bioaccumulation of manganese and nickel.	[232,244]
*R. delica*	-	- Vinegar produced via microwave-assisted enzymatic hydrolysis with high nutritional value and antioxidant activity.	[240]
*R. foetens*	Hg: 0.06 mg/kg	- Lowest Hg level among analyzed species.	[227]
*R. nigricans*	DCAA: 23–37 mg/kg	- DCAA present, suggesting biosynthesis in this species, challenging traditional views of DCAA as a pollutant.	[243]
*R. ochroleuca*	Hg: 0.017 to 0.43 μg/g (caps), 0.011 to 0.24 μg/g (stipes)	- Caps had higher Hg concentrations. - Potential bioindicator for environmental Hg pollution.	[118]
*R. virescens*	-	- Purified novel laccase with 69 kDa; optimal activity at pH 2.2 and 60 °C. - Degrades phenolic compounds and decolorizes dyes.	[242]
*Russula* species	Fe, Zn, Cu (varied concentrations)	- Can accumulate and translocate heavy metals under natural pH conditions, with significant mobility within fruiting bodies.	[239]
	Zn: high concentrations	- Substantial contributions to metal cycling and environmental sequestration.	[233,234,235,236,237,238]

## Data Availability

No new data were produced.
